# Effect of obesity on perioperative outcomes following lung cancer surgery: a systematic review and meta-analysis

**DOI:** 10.3389/fonc.2025.1600503

**Published:** 2025-09-25

**Authors:** Qiuxiang Wang, Zhishu Li, Xi Huan Wang, Bin Li, Chunfeng Wang, Yongguo Xiang

**Affiliations:** ^1^ Department of Traditional Chinese Medicine, Guangyuan Central Hospital, Guangyuan, Sichuan, China; ^2^ Department of Respiratory Medicine,Guangyuan Central Hospital, Guangyuan, Sichuan, China

**Keywords:** obesity, lung cancer, perioperative outcomes, surgery, meta-analysis

## Abstract

**Background:**

This meta-analysis systematically evaluated the impact of obesity on perioperative outcomes in patients undergoing lung cancer surgery to provide robust evidence for clinical decision-making and patient management.

**Methods:**

A systematic review was conducted in accordance with Preferred Reporting Items for Systematic Reviews and Meta-analyses guidelines. Comprehensive searches were performed across PubMed, Embase, The Cochrane Library, Web of Science, and Medline. A random-effects model was employed, and the meta-analysis was conducted using Stata software (version 16.0).

**Results:**

Eighteen studies comprising 88,413 participants were included in the meta-analysis. Postoperative mortality was significantly lower in the obese group (odds ratio [OR]: 0.73; 95% confidence interval [CI]: 0.59–0.90; *P* = 0.003). Statistically non-significant differences were observed between the two groups regarding postoperative complications (OR: 1.14; 95% CI: 0.71–1.84; *P* = 0.582), pulmonary complications (OR: 1.21; 95% CI: 0.57–2.58; *P* = 0.621), cardiovascular complications (OR: 1.54; 95% CI: 0.86–2.76, *P* = 0.150), and the total hospital stays (standardized mean difference [SMD]: −0.00; 95% CI: −0.17 to 0.17; *P* = 0.995). However, operation time was significantly longer in the obese group (SMD: 0.18; 95% CI: 0.11–0.26; *P* < 0.001).

**Conclusion:**

The obesity paradox may exist in patients undergoing lung cancer surgery. Although obesity is associated with longer operation duration, it neither increases the risk of complications nor prolongs hospital stays and is generally linked to reduced postoperative mortality.

**Systematic review registration:**

https://www.crd.york.ac.uk/PROSPERO/view/CRD42025648330, identifier CRD42025648330

## 1 Introduction

Lung cancer remains the leading cause of cancer-related mortality globally. Surgical resection constitutes the primary curative treatment for early-stage disease (1). In 2022, lung cancer accounted for nearly 2.5 million new cases and over 1.8 million deaths worldwide, comprising approximately 12.4% of global cancer diagnoses and 18.7% of cancer-related deaths (2). Given its high incidence and mortality, lung cancer diagnosis and treatment have long been the subject of extensive investigation. Histologically, it is classified into two main types: small-cell lung cancer (SCLC) and non-small-cell lung cancer (NSCLC) (3). SCLC frequently presents as a disseminated disease and is primarily treated with systemic therapy. Surgical intervention is reserved for carefully selected patients with limited-stage disease, typically defined as a solitary pulmonary nodule with regional lymph node involvement and no distant metastases (4). In contrast, surgical resection remains the main treatment for early-stage NSCLC. However, it carries considerable perioperative risks, including respiratory complications, cardiovascular events, and mortality (5).

The World Health Organization (WHO) defines overweight as a body mass index (BMI) greater than 25 kg/m^2^ and obesity as a BMI exceeding 30 kg/m^2^ (6). Obesity is regarded as a major global public health concern, with its prevalence having doubled in 70 countries since 1980 and increased in nearly all other countries (7). The global obesity epidemic—driven by sedentary lifestyles, poor dietary habits, genetic predisposition, and environmental influences—has resulted in over 1.9 billion adults being classified as overweight and 650 million as obese (8). Currently, more than one-third of the global population falls into these categories, with projections indicating that by 2030, 38% of adults will be overweight and 20% will be obese (9). This rising burden presents specific challenges for surgeons, particularly in managing the physical, technical, and physiological complexities of thoracic operation in patients with obesity.

Obesity is a major contributor to the development of established cardiovascular risk factors, including diabetes, hypertension, dyslipidemia, and chronic kidney disease (10). It also constitutes a significant risk factor for lung cancer, influencing multiple aspects of the disease. Obesity contributes to tumor onset and progression through mechanisms such as chronic inflammation, hormonal imbalances, and metabolic dysregulation (9). Additionally, obesity complicates clinical outcomes, often resulting in poorer prognoses, increased treatment-related complications, and a higher incidence of comorbidities (9). Despite its established association with comorbid conditions, the impact of obesity on perioperative outcomes in lung cancer surgery remains controversial. Whether patients with obesity experience higher incidences of postoperative complications compared to individuals with normal weight undergoing lung cancer surgery continues to be a subject of investigation.

Some studies suggest that obesity may elevate the risk of postoperative complications, such as respiratory complications, due to physiological stress and technical challenges during surgery (11). Conversely, other studies support the existence of an “obesity paradox,” wherein patients with a BMI of ≥ 30 kg/m^2^ demonstrate improved survival and fewer complications compared to those of normal weight or underweight following lung cancer resection (12). This discrepancy highlights the need for a comprehensive synthesis of existing evidence.

Li S et al. conducted a systematic review examining the prognostic significance of BMI in patients undergoing lung cancer surgery, with a particular emphasis on the “obesity paradox” (13). This paradox refers to the counterintuitive observation that obese patients may experience improved survival compared to those with normal or low BMI. The review concluded that higher BMI and obesity are associated with favorable in-hospital outcomes and enhanced long-term survival in patients undergoing lung cancer surgery, indicating that the obesity paradox may exist in this patient population. However, the authors noted that potential ethnic variations and confounding variables need to warrant investigation. Notably, the study did not comprehensively evaluate the impact of obesity on key perioperative outcomes, including operation time, postoperative complications, and mortality. Consequently, the conclusions of the previous review offer limited utility for preoperative risk stratification and the optimization of preoperative care. Furthermore, several recent studies published after 2016 have investigated the impact of obesity on perioperative outcomes following lung cancer surgery. To address this, a meta-analysis was conducted to systematically assess the influence of obesity on perioperative outcomes in patients undergoing lung cancer resection, with the objective of providing stronger evidence to inform clinical decision-making and patient management.

## 2 Methods

### 2.1 Study registration

This meta-analysis was conducted in accordance with the Preferred Reporting Items for Systematic Reviews and Meta-analyses (PRISMA) 2020 guidelines (14) and registered with PROSPERO (registration number: CRD42025648330). The PRISMA checklist is provided in **Supplementary Material 1**.

### 2.2 Selection criteria

#### 2.2.1 Types of studies

This meta-analysis included observational studies, specifically cross-sectional, case-control, and cohort studies. Case reports, case series, review articles, letters, editorials, and commentaries were excluded.

#### 2.2.2 Types of participants

Included participants were obese patients aged 18 years or older with a diagnosis of lung cancer who underwent surgical resection. Obesity was defined according to WHO criteria: a BMI > 30 kg/m^2^ for Western populations and > 25 kg/m^2^ for Asian populations.

#### 2.2.3 Types of interventions and comparisons

Eligible interventions included video-assisted thoracoscopic surgery (VATS), robotic-assisted thoracoscopic surgery, open lobectomy, open pneumonectomy, open wedge resection, and open segmental resection. The control group comprised non-obese patients diagnosed with lung cancer who underwent surgical resection.

#### 2.2.4 Language

Only studies published in English were included in the meta-analysis.

### 2.3 Exclusion criteria

Studies were excluded if they met any of the following criteria:

1. Published in languages other than English.

2. Did not report standardized incidence ratios, odds ratios (OR), risk ratios, hazard ratios (with 95% confidence interval [CI]), standard errors, or event counts necessary for effect size calculation.

3. Did not include a non-obese comparison group.

4. Contained discrepancies between textual data and tabulated results.

### 2.4 Search strategy

A comprehensive systematic search was performed across five databases—PubMed, Embase, The Cochrane Library, Web of Science, and Medline—from inception to January 2025 to identify eligible studies. The search strategy employed Medical Subject Headings (MeSH) and relevant keywords: [(lung cancer OR lung neoplasm) AND (VATS OR pulmonary surgical procedures) AND (BMI OR obesity OR obese)]. PubMed search strategy was used as a representative example, with detailed retrieval steps provided in **Supplementary Material 2**. Additional sources, including journal articles, conference papers, and academic papers, were also examined.

### 2.5 Data collection and analysis

#### 2.5.1 Study selection

**Figure 1:**
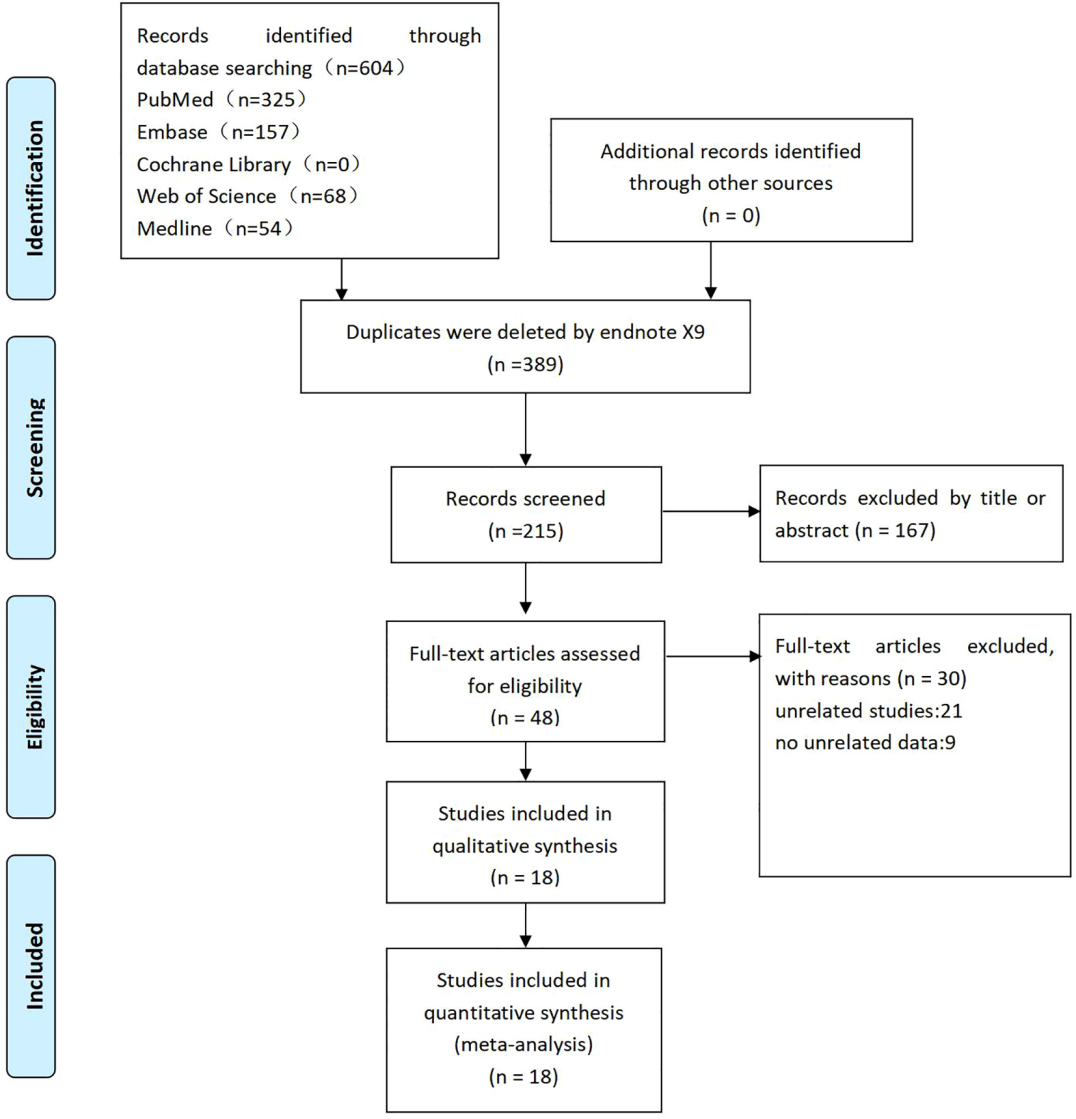
PRISMA flow chart.

Two independent reviewers (CW and XW) screened the literature based on the predefined inclusion and exclusion criteria. Duplicate records were identified and removed using EndNoteX9 software, and abstracts and keywords were reviewed to eliminate irrelevant studies. Eligible studies were included in the meta-analysis after full-text assessment. Discrepancies were resolved through group discussion, and unresolved conflicts were adjudicated by the corresponding author (YX). The complete study selection process is illustrated in a PRISMA flow diagram (**Figure 1**).

#### 2.5.2 Data extraction

Two reviewers independently extracted data from the included studies and entered them into a predefined Excel-based data extraction form. Discrepancies were resolved through discussion, and unresolved conflicts were arbitrated by the corresponding author (YX). Extracted data included the following: Study characteristics: first author’s name, year of publication, country, sample size, patient age, surgical approach, and tumor staging. Primary outcomes: postoperative mortality and postoperative complications. Secondary outcomes: pulmonary complications, cardiovascular complications, total hospital stay, and operation time.

#### 2.5.3 Quality assessment

The quality of the included studies was evaluated using the Newcastle–Ottawa Scale (NOS) (15). This tool evaluates the quality of reporting and the risk of bias in included studies using a structured checklist covering three domains: selection, comparability, and exposure or outcomes (15). The scale consists of eight items under three parameters, with a total score of 9. Studies scoring ≤ 3 were classified as low quality, 4–5 as medium quality, and ≥ 6 as high quality (16). Two reviewers (QW and CW) independently assessed the quality of the included studies, and a third reviewer (YX) verified the results. Discrepancies were resolved through discussion.

#### 2.5.4 Data analysis and heterogeneity processing

Meta-analysis and statistical analysis were conducted using Stata software (version 16.0). Given the variability in patients’ risk profiles and surgical selection criteria across different centers, a random effects model was applied to account for heterogeneity and produce more conservative estimates (17, 18). When outcomes were reported as medians with interquartile ranges, the method described by Wan et al. was employed to estimate the mean and standard deviation (19, 20). Dichotomous outcomes are presented as OR with 95% CIs, while continuous variables are expressed as standardized mean difference (SMD) with 95% CIs. Statistical significance was defined as *P* < 0.05. Heterogeneity was significant when I^2^ > 50% and/or *P* < 0.1. Additionally, levels of heterogeneity, assessed by I^2^ statistic, were interpreted as follows: 0%–25% indicated homogeneity, 25%–50% low heterogeneity, 50%–75% moderate heterogeneity, and > 75% high heterogeneity (21). Sensitivity analysis was performed by omitting one study at a time and recalculating the summary data from the remaining studies to evaluate the impact of each included study on the overall results. Publication bias was assessed using Egger’s test and funnel plots when ≥ 10 studies were available (22).

## 3 Results

### 3.1 Literature search

A literature search of five electronic databases was conducted to identify English-language studies examining the effect of obesity on perioperative outcomes following lung cancer surgery. Relevant studies were imported into EndNoteX9 software, and duplicates were removed, resulting in 247 records. Of these, 167 studies were excluded based on titles or abstracts. After a thorough full-text evaluation, 18 studies met the eligibility criteria for inclusion in the meta-analysis.

### 3.2 Characteristics of studies included in the meta-analysis

Eighteen studies were included in the meta-analysis, comprising 88,413 participants: 14,050 obese and 74,363 normal-weight patients who underwent lung cancer surgery. Six studies were conducted in the United States of America (23–28), four in Italy (11, 29–31), two in China (32, 33), two in Japan (34, 35), and two in the Czech Republic (12, 36). The remaining studies were performed in the United Kingdom (37) and France (38). Baseline characteristics demonstrated statistically non-significant differences between obese and normal-weight groups. The characteristics of the included studies are summarized in **Table 1**, and the study outcomes are presented in **Table 2**.

**Table 1:** The detailed characteristics of included studies.

Author/ year	Country	Study design	Study period	Sample size	Age: mean±SD/ mean(min-max)/ median (IQR)/ >65, n (%)/mean years	Gender Male/Female	Stages	Approaches
Vadlakonda A et al.2023 (23)	USA	retrospective cohort	2012-2020	Normal: 13675 Obesity: 6424	Normal 50.3±9.7 Obesity: 49.3±9.4	Normal:6263/7412 Obesity:2852/3572	I–IV	wedge resection, lobectomy, pneumonectomy
Smith, Philip W et al.2007 (24)	USA	retrospective cohort	2002-2006	Normal:372 Obesity:127	Normal:66.5 Obesity:66.4	Normal:212/160 Obesity:61/66	I–IV	Segmentectomy Lobectomy Bilobectomy Pneumonectomy VATS Chest wall resection
Montané, Bryce et al.2017 (25)	USA	retrospective cohort	2010-2015	Normal:94 Obesity:80	Normal:68±1.0 Obesity:67±1.1	Normal:38/56 Obesity:40/40	I–IV	Robotic-Assisted Pulmonary Lobectomy
Dhakal, Binod et al. 2013 (26)	USA	retrospective cohort	2006-2010	Normal:121 Obesity:199	Normal:67(25-88) Obesity:67(44-85)	Normal:49/72 Obesity:86/113	I–IV	Lobectomy Wedge Resection Pneumonectomy
Launer, Hunter et al.2013 (27)	USA	retrospective cohort	2002-2007	Normal:31983 Obesity:1238	Normal:66.7±10.5 Obesity:64.8±9.9	Normal:15965/15987 Obesity:501/737	I–IV	pulmonary lobectomy
Mungo, Benedetto et al.2015 (28)	USA	retrospective cohort	2005-2012	Normal:2163 ObeseI:1244 ObeseII:455 ObeseIII:213	Normal:68(60-75) ObeseI:68(61-74) ObeseII:67(60-72) ObeseIII:65(57-70)	Normal:917/1247 ObeseI:650/594 ObeseII:204/251 ObeseIII:84/129	I–IV	pulmonary resections
Guerrera, Francesco et al.2022 (29)	Italy	retrospective cohort	2016-2019	Normal:4338 Obesity:74	Normal:69 (63–75) Obesity:68 (59–63)	Normal:2641/1697 Obesity:40/34	I–IV	VATS
Leonardi, Beatrice et al. 2023 (30)	Italy	retrospective cohort	2019-2022	Normal:85 Obesity:26	Normal:66.8±7.8 Obesity:64.5±7.4	Normal:56/29 Obesity:12/14	I–IV	thoracoscopic lobectomy
Petrella, Francesco et al. 2011 (11)	Italy	retrospective cohort	2004-2008	Normal:61 Obesity:93	Normal:63.8±10.2 Obesity:63.3±8.9	Normal:44/17 Obesity:80/13	I–IV	Standard pneumonectomy
Zirafa, Carmelina Cristina et al.2021 (31)	Italy	retrospective cohort	2010-2017	Normal:50 Obesity:34	NR	NR	III-IV	robotic assisted lung resection
Tong, Chaoyang et al.2022 (32)	China	retrospective cohort	2016-2018	Normal:3730 Obesity:305	Normal:69.8±4.1 Obesity:69.5±3.9	Normal:1748/1982 Obesity:137/168	I–IV	VATS RATS VATS:Video-assisted thoracoscopic surgery; RATS: Robotic-assisted thoracoscopic surgery
Li, Rongyang et al.2022 (33)	China	retrospective cohort	2020-2021	RAL:292 Normal:113 Obesity:179 VAL:292 Normal:105 Obesity:187	RAL:58(52-65) VAL:60(53-66)	RAL:172/187 VAL:197/266	I–IV	robotic-assistedlobectomy(RAL) video-assistedlobectomy(VAL)
Nakagawa, Tatsuo et al.2016 (34)	Japan	retrospective cohort	2001-2011	Normal:940 Obesity:25	Normal:68.2±8.6 Obesity:66.6±5.9	Normal:626/314 Obesity:10/15	I–III	Pneumonectomy Lobectomy Less than lobectomy
Matsunaga, Takeshi et al. 2015 (35)	Japan	retrospective cohort	2008-2013	Normal:1037 Obesity:26	Normal:67(14-90) Obesity:64(25-83)	Normal:592/445 Obesity:14/12	I–IV	Pneumonectomy Lobectomy Segmentectomy Wide wedge resection
Tulinský, Lubomír et al. 2018 (36)	Czech Republic	retrospective cohort	2014-2016	Normal:133 Obesity:70	Normal:60.0±9.2 Obesity:65.2±8.9	Normal:82/51 Obesity:44/26	I–IV	pulmonary lobectomy Thoracoscopy Thoracotomy
Tulinský, Lubomír et al. 2022 (12)	Czech Republic	retrospective cohort	2016-2018	Normal:96 Obesity:48	Normal:63.5±9.4 Obesity:64.7±7.9	Normal:63/33 Obesity:29/19	I–IV	lung lobectomy Thoracoscopy Thoracotomy
Attaran, Saina et al.2012 (37)	UK	retrospective cohort	2000-2010	Normal:1550 Obesity:337	Normal:69(62-75) Obesity:67(60-74)	Normal:819/731 Obesity:180/157	I–III	Wedge resection Lobectomy Pneumonectomy
Thomas, Pascal Alexandre et al.2013 (38)	France	retrospective cohort	2005-2011	Normal:9391 Obesity:2666	Normal:3595 Obesity:1262	Normal:6392/2999 Obesity:1978/688	early I–II, Locally advanced III, metastatic IV	VATS Upper lobectomy Extended lobectomy Radical lymphadenectomy

VATS, video-assisted thoracoscopic surgery.

**Table 2:** The outcomes of each included study.

Author	Operation time (min) mean±SD /median (IQR)	Postoperative mortality	Pulmonary complications	Surgical complications	Cardiovascular complications	Hospital length of stay
Vadlakonda,Amulya et al. 2023 (23)	Normal:182±89 Obesity:189±88	Normal:NR Obesity:NR	Normal:NR Obesity:NR	Normal:NR Obesity:NR	Normal:NR Obesity:NR	Normal:.7±4.9 Obesity:5.2±4.3
Smith, Philip W et al.2007 (24)	Normal:NR Obesity:NR	Normal:5 Obesity:2	Normal:81 Obesity:18	Normal:124 Obesity:39	Normal:45 Obesity:21	Normal:NR Obesity:NR
Montané, Bryce et al.2017 (25)	Normal:178±9 Obesity:188±8	Normal:1 Obesity:3	Normal:26 Obesity:20	Normal:31 Obesity:27	Normal:13 Obesity:14	Normal:5±0.4 Obesity:4±0.6
Dhakal, Binod et al. 2013 (26)	Normal:NR Obesity:NR	Normal:4 Obesity:2	Normal:15 Obesity:21	Normal:28 Obesity:47	Normal:11 Obesity:24	Normal:NR Obesity:NR
Launer, Hunter et al.2013 (27)	Normal:NR Obesity:NR	Normal:NR Obesity:NR	Normal:1858 Obesity:445	Normal:NR Obesity:NR	Normal:1204 Obesity:246	Normal:NR Obesity:NR
Mungo, Benedetto et al.2015 (28)	Normal:160(119-215) ObeseI:170(129-228) ObeseII:168(125-227) ObeseIII:174(126-228)	Normal:63 ObeseI:25 ObeseII:8 ObeseIII:3	Normal:2 ObeseI:0 ObeseII:1 ObeseIII:0	Normal:361 ObeseI:354 ObeseII:196 ObeseIII:77	Normal:28 ObeseI:24 ObeseII:6 ObeseIII:2	Normal:6(4-9) ObeseI:5(4-7) ObeseII:6(4-8) ObeseIII:7(5-9)
Guerrera, Francesco et al.2022 (29)	Normal:NR Obesity:NR	Normal:NR Obesity:NR	Normal:NR Obesity:NR	Normal:880 Obesity:26	Normal:NR Obesity:NR	Normal:NR Obesity:NR
Leonardi, Beatrice et al. 2023 (30)	Normal:297±78 Obesity:296±69	Normal:2 Obesity:1	Normal:60 Obesity:20	Normal:NR Obesity:NR	Normal:5 Obesity:2	Normal:7.6±4.2 Obesity:7.2±4.1
Petrella, Francesco et al. 2011 (11)	Normal:NR Obesity:NR	Normal:3 Obesity:7	Normal:3 Obesity:20	Normal:34 Obesity:57	Normal:14 Obesity:28	Normal:NR Obesity:NR
Zirafa, Carmelina Cristina et al.2021 (31)	Normal:NR Obesity:NR	Normal:NR Obesity:NR	Normal:NR Obesity:NR	Normal:32 Obesity:21	Normal:NR Obesity:NR	Normal:7(4-23) Obesity:6(4-23)
Tong, Chaoyang et al.2022 (32)	Normal:102.4±39.5 Obesity:108.5±40.1	Normal:19 Obesity:2	Normal:1309 Obesity:118	Normal:241 Obesity:32	Normal:174 Obesity:17	Normal:NR Obesity:NR
Li, Rongyang et al.2022 (33)	RAL: Normal:90(70-110) Obesity:95(80-120) VAL: Normal:105(80-125) Obesity:120(90-140)	RAL: Normal:NR Obesity:NR VAL: Normal:NR Obesity:NR	RAL: Normal:NR Obesity:NR VAL: Normal:NR Obesity:NR	RAL: Normal:35 Obesity:32 VAL: Normal:26 Obesity:50	RAL: Normal:NR Obesity:NR VAL: Normal:NR Obesity:NR	RAL: Normal:5(4-8) Obesity:5(4-6) VAL: Normal:5(4-7) Obesity:5(4-6)
Nakagawa, Tatsuo et al.2016 (34)	Normal:NR Obesity:NR	Normal:NR Obesity:NR	Normal:186 Obesity:6	Normal:NR Obesity:NR	Normal:6 Obesity:3	Normal:NR Obesity:NR
Matsunaga, Takeshi et al. 2015 (35)	Normal:145(28-482) Obesity:140(42-325)	Normal:6 Obesity:0	Normal:87 Obesity:3	Normal:221 Obesity:5	Normal:91 Obesity:1	Normal:7(3-113) Obesity:8(4-17)
Tulinský, Lubomír et al. 2018 (36)	Normal:95.2±30.9 Obesity:105.5±32.2	Normal:5 Obesity:0	Normal:NR Obesity:NR	Normal:45 Obesity:19	Normal:NR Obesity:NR	Normal:11.3±6.3 Obesity:10.5±5.7
Tulinský, Lubomír et al. 2022 (12)	Normal:98.3±31.2 Obesity:107.1±33.5	Normal:4 Obesity:0	Normal:NR Obesity:NR	Normal:33 Obesity:13	Normal:NR Obesity:NR	Normal:11.5±6.4 Obesity:9.8±5.3
Attaran, Saina et al.2012 (37)	Normal:NR Obesity:NR	Normal:35 Obesity:8	Normal:NR Obesity:NR	Normal:NR Obesity:NR	Normal:NR Obesity:NR	Normal:7(6-10) Obesity:7(6-9)
Thomas, Pascal Alexandre et al.2013 (38)	Normal:133±56 Obesity:134±55	Normal:249 Obesity:50	Normal:1369 Obesity:402	Normal:1293 Obesity:188	Normal:513 Obesity:193	Normal:NR Obesity:NR

NR, not reported; SD, standard deviation; IQR, interquartile range.

### 3.3 Quality assessment of the included studies

Based on the NOS, one study received six stars, four received seven stars, 10 received eight stars, and the remaining three received nine stars. All included studies were of high quality. Quality assessments according to the NOS are presented in **Table 3**.

**Table 3:** Quality assessment of included studies.

Study ID	Selection (Out of 4)				Comparability (Out of 2)	Outcomes (Out of 3)			Total
	①	②	③	④		⑤	⑥	⑦	
Vadlakonda A,2023[23]	*	*	*	*	*	*	*	*	8
Smith PW,2007[24]	*	*	*	*	*	*	*	*	8
Montané B,2017[25]	*	*	*	*	*	*	**-**	**-**	7
Dhakal B,2013[26]	*	*	*	*	*	*	*	*	8
Launer H,2013[27]	*	*	*	*	**	*	**-**	**-**	7
Mungo B,2015[28]	*	*	*	*	*	*	*	*	8
Guerrera F,2022[29]	*	*	*	*	**	*	**-**	**-**	7
Leonardi B,2023[30]	*	*	*	*	*	*	*	*	8
Petrella F,2011[11]	*	*	*	*	*	*	*	*	8
Zirafa CC,2021[31]	*	*	*	*	*	*	–	–	6
Tong C,2022[32]	*	*	*	*	*	*	*	*	8
Li R,2022[33]	*	*	*	*	**	*	–	–	7
Nakagawa T,2016[34]	*	*	*	*	**	*	*	*	9
Matsunaga T,2015[35]	*	*	*	*	*	*	*	*	8
Tulinský L,2018[36]	*	*	*	*	*	*	*	*	8
Tulinský L,2022[12]	*	*	*	*	*	*	*	*	8
Attaran S,2012[37]	*	*	*	*	**	*	*	*	9
Thomas PA,2013[38]	*	*	*	*	**	*	*	*	9

①representativeness of exposed cohort; ②selection of non-exposed cohort; ③ascertainment of exposure; ④outcome not present at the start of the study; ⑤assessment of outcomes; ⑥length of follow-up; ⑦ adequacy of follow-up.

The symbol *and ** indicate the use of the Newcastle-Ottawa scale to evaluate the quality of included studies.

### 3.4 Primary surgical outcomes

#### 3.4.1 Postoperative 30-day mortality

**Figure 2:**
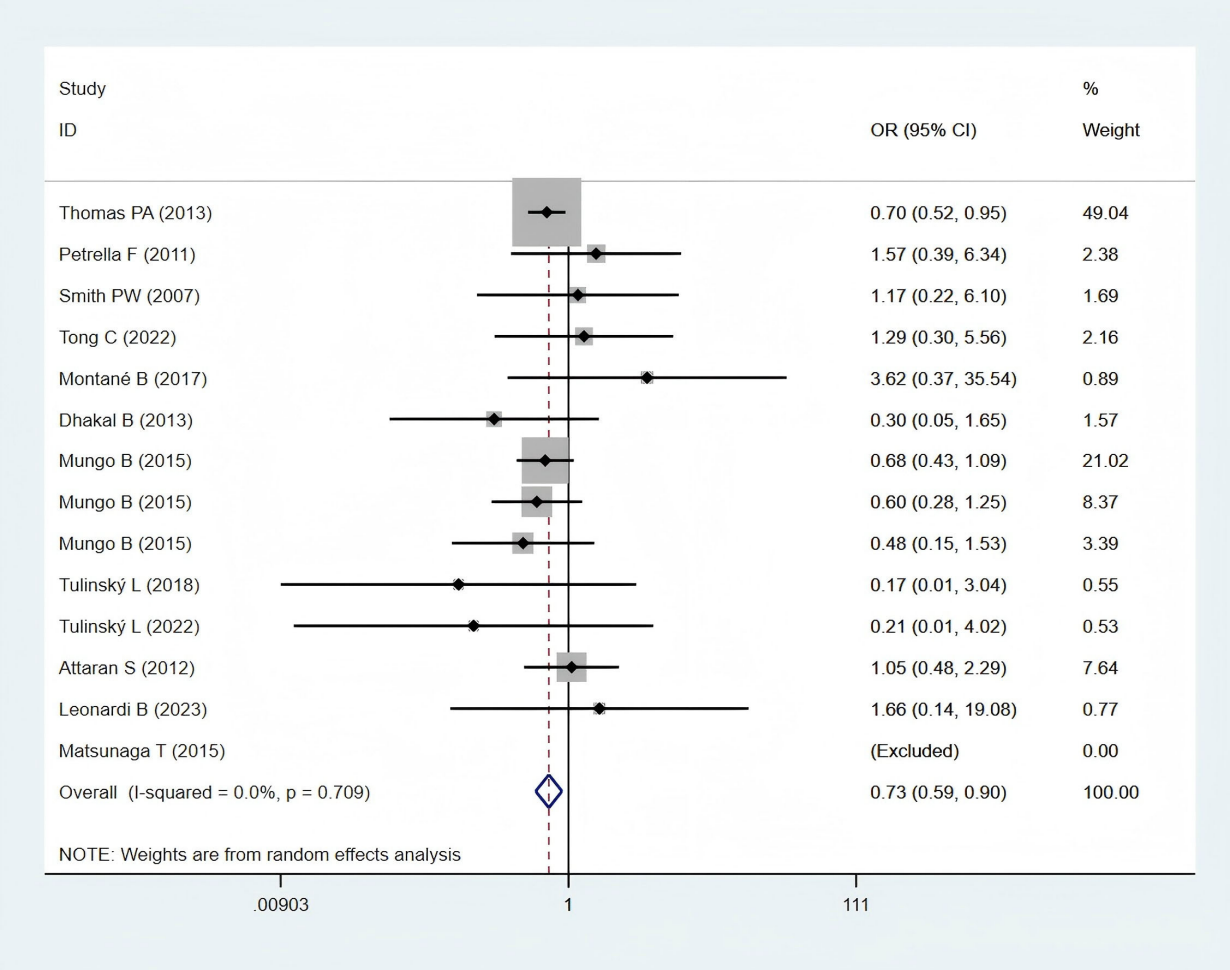
Forest plots for postoperative 30-day mortality between obese groups and normal groups.

Twelve studies (11, 12, 24–26, 28, 30, 32, 35–38), encompassing 5,889 obese and 23,159 normal-weight patients, reported data on postoperative 30-day mortality and were included in the meta-analysis. A statistically significant difference in postoperative 30-day mortality was observed between the two groups (OR: 0.73; 95% CI: 0.59–0.90; *P* = 0.003; I² = 0.0%, *P* = 0.709) (**Figure 2**), with significantly lower mortality in the obese group.

#### 3.4.2 Postoperative complications

**Figure 3:**
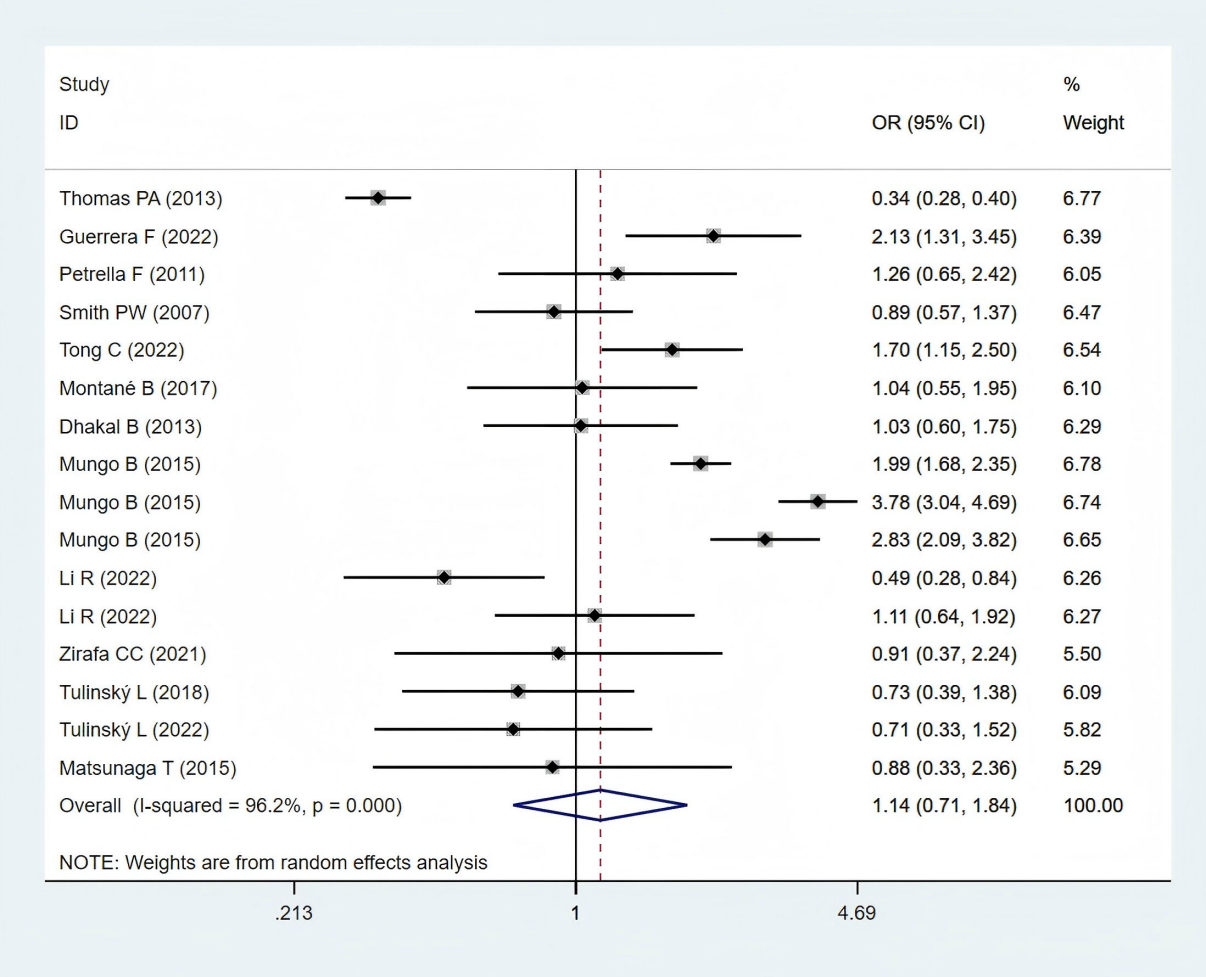
Forest plots for postoperative complications between obese groups and normal groups.

Thirteen studies (11, 12, 24–26, 28, 29, 31–33, 35, 36, 38), comprising 6,000 obese and 26,130 normal-weight patients, reported postoperative complication data and were included in the meta-analysis. A statistically non-significant difference in postoperative complications was observed between the two groups (OR: 1.14; 95% CI: 0.71–1.84; *P* = 0.582; I² = 96.2%, *P* < 0.001) (**Figure 3**).

### 3.5 Secondary surgical outcomes

#### 3.5.1 Respiratory complications

**Figure 4:**
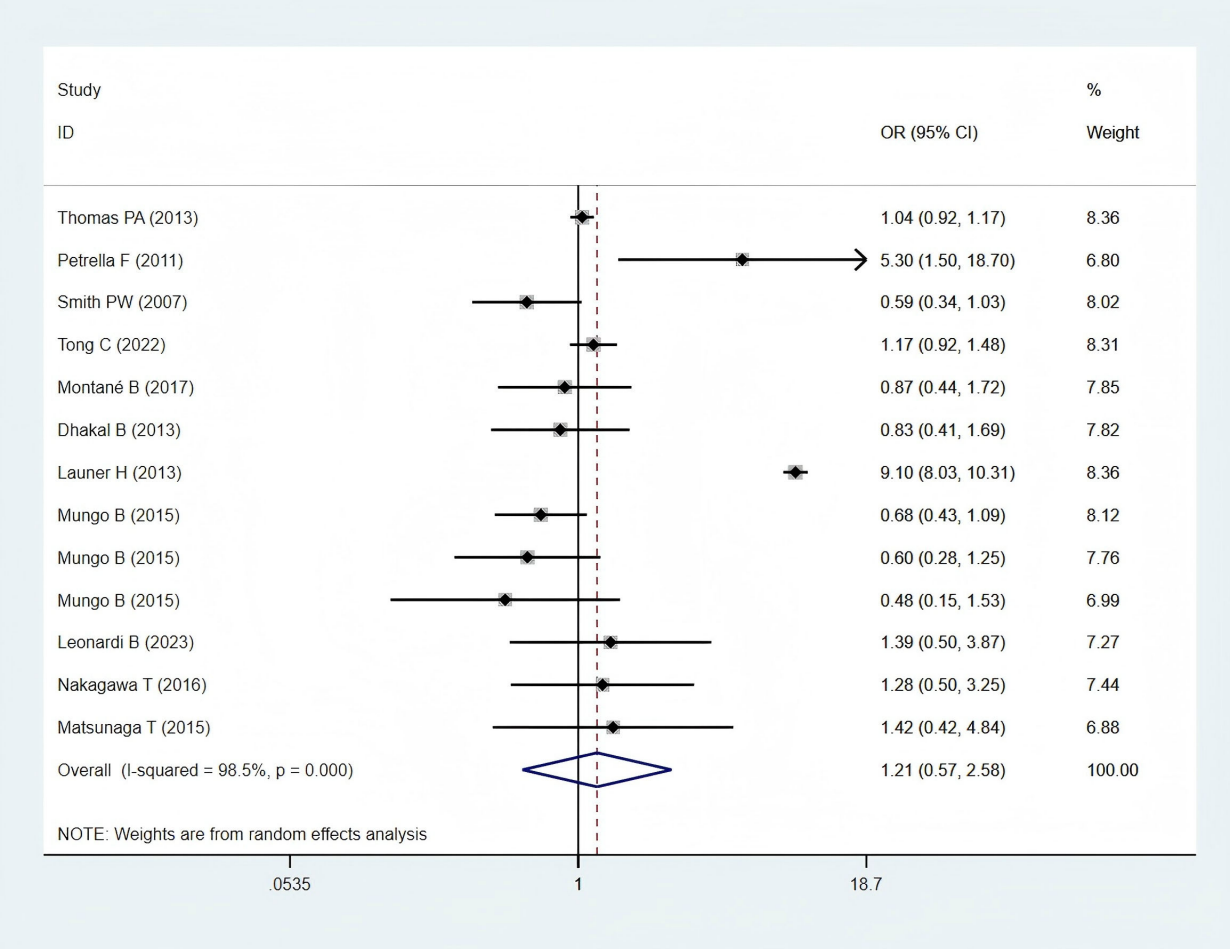
Forest plots for respiratory complications between obese groups and normal groups.

Eleven studies (11, 24–28, 30, 32, 34, 35, 38), including 6,697 patients in the obese group and 54,303 patients in the normal-weight group, provided respiratory complication data and were included in the meta-analysis. Respiratory complications, including lung infection, air leakage, acute respiratory distress syndrome, empyema, pneumonia, pulmonary embolism, pulmonary insufficiency, respiratory failure and other respiratory system complications. A statistically non-significant difference in pulmonary complications was found between the two groups (OR: 1.21; 95% CI: 0.57–2.58; *P* = 0.621; I² = 98.5%, *P* < 0.001) (**Figure 4**).

#### 3.5.2 Cardiovascular complications

**Figure 5:**
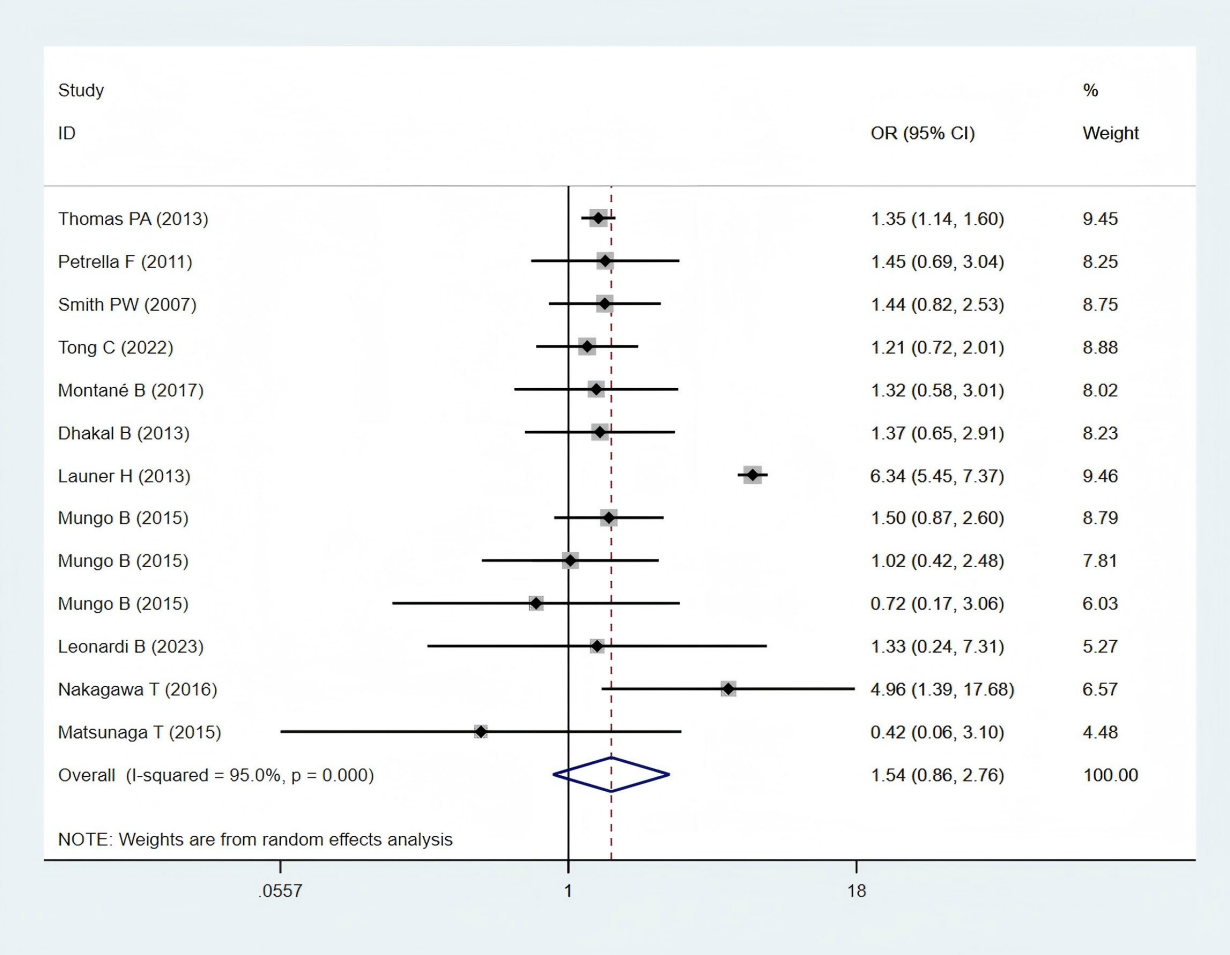
Forest plots for cardiovascular complications between obese groups and normal groups.

Eleven studies (11, 24–28, 30, 32, 34, 35, 38), comprising 6,697 patients in the obese group and 54,303 patients in the normal-weight group, reported cardiovascular complication data and were included in the meta-analysis. Cardiovascular complications, including arrhythmia, cardiac arrest, myocardial infarction, atrial fibrillation, other arrhythmia and other cardiac complications. A statistically non-significant difference in cardiovascular complications was observed between the two groups (OR: 1.54; 95% CI: 0.86–2.76; *P* = 0.150; I² = 95.0%, *P* < 0.001) (**Figure 5**).

#### 3.5.3 Total hospital stay

**Figure 6:**
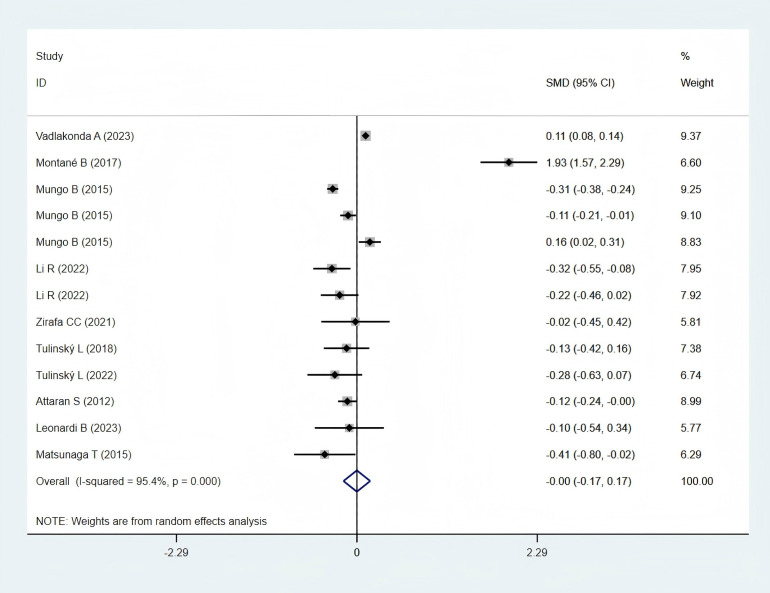
Forest plots for total hospital stays between obese groups and normal groups.

Ten studies (12, 23, 25, 28, 30, 31, 33, 35–37), comprising 9,323 patients in the obese group and 23,427 in the normal-weight group, reported data on total hospital stays and were included in the meta-analysis. A statistically non-significant difference was observed in total hospital stays between the two groups (SMD: −0.00; 95% CI: −0.17 to 0.17; *P* = 0.995; I² = 95.4%, *P* < 0.001) (**Figure 6**).

#### 3.5.4 Operation time

**Figure 7:**
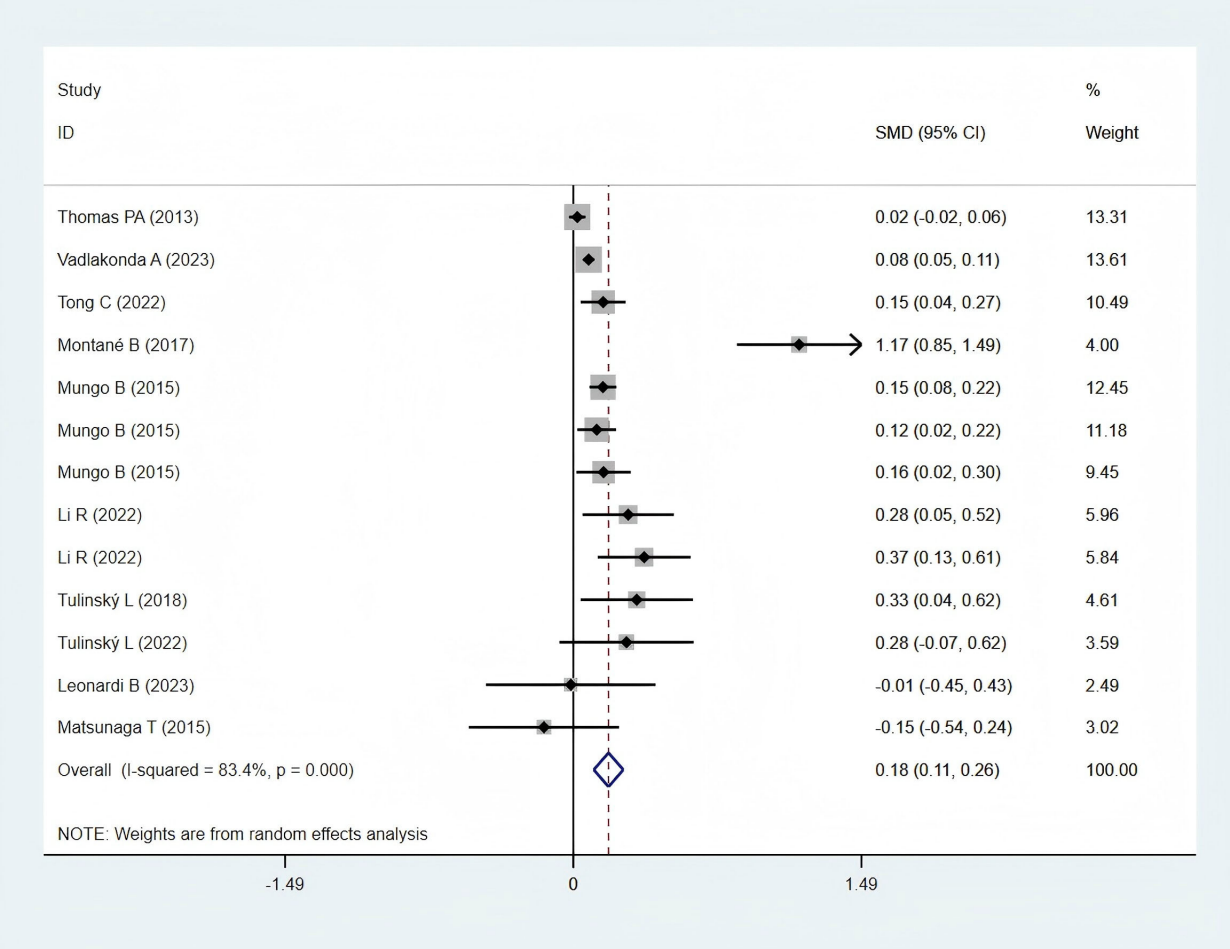
Forest plots for operation time between obese groups and normal groups.

Ten studies (12, 23, 25, 28, 30, 32, 33, 35, 36, 38), including 11,923 patients in the obese group and 32,948 in the normal-weight group, reported operation time data and were included in the meta-analysis. Operation time was significantly longer in the obese group (SMD: 0.18; 95% CI: 0.11–0.26; *P* < 0.001; I² = 84.3%, *P* < 0.001) (**Figure 7**).

### 3.6 Subgroup analysis

#### 3.6.1 Subgroup analysis of postoperative 30-day mortality

**Figure 8:**
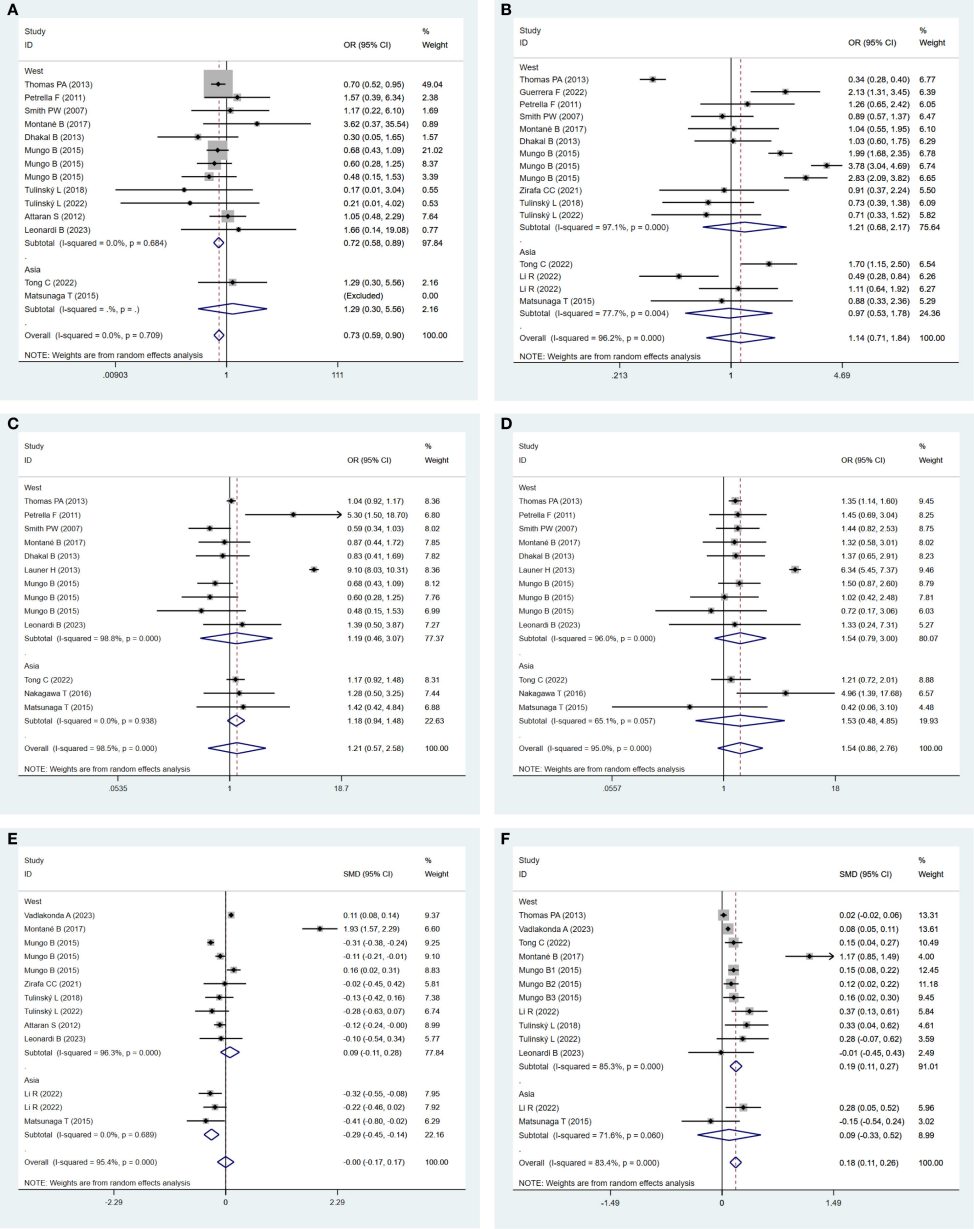
**(A)** Forest plots for subgroup analysis of postoperative 30-day mortality between obese groups and normal groups. **(B)** Forest plots for subgroup analysis of postoperative complications between obese groups and normal groups. **(C)** Forest plots for subgroup analysis of respiratory complications between obese groups and normal groups. **(D)** Forest plots for subgroup analysis of cardiovascular complications between obese groups and normal groups. **(E)** Forest plots for subgroup analysis of total hospital stay between obese groups and normal groups. **(F)** Forest plots for subgroup analysis of operation time between obese groups and normal groups.

Subgroup analysis demonstrated that postoperative 30-day mortality was significantly lower in the obesity group in the Western populations (OR: 0.72; 95% CI: 0.58–0.89; *P* = 0.003; I² = 0.0%, *P* = 0.684). In contrast, no significant difference was observed between the groups in Asian populations (**Figure 8A**).

#### 3.6.2 Subgroup analysis of postoperative complications

Subgroup analysis indicated a statistically non-significant difference in postoperative complications between the obese and normal-weight groups, either in Western populations (OR: 1.21; 95% CI: 0.68–2.17, *P* = 0.52; I² = 97.1%, *P* = 0.000) or in Asian populations (OR: 0.97; 95% CI: 0.53–1.78, *P* = 0.924; I² = 77.1%, *P* = 0.004) (**Figure 8B**).

#### 3.6.3 Subgroup analysis of respiratory complications

Subgroup analysis revealed a statistically non-significant difference in respiratory complications between the obese and normal-weight groups, either in Western populations (OR: 1.19; 95% CI: 0.46–3.07; *P* = 0.717; I² = 98.8%, *P* = 0.000) or in Asian populations (OR: 1.18; 95% CI: 0.94–1.48; *P* = 0.152; I² = 0.0%, *P* = 0.938) (**Figure 8C**).

#### 3.6.4 Subgroup analysis of cardiovascular complications

Subgroup analysis indicated a statistically non-significant difference in cardiovascular complications between the obese and normal-weight groups, either in Western populations (OR: 1.54; 95% CI: 0.79–3.00; *P* = 0.205; I² = 96.0%, *P* = 0.000) or in Asian populations (OR: 1.53; 95% CI: 0.48–4.85; *P* = 0.469; I² = 65.1%, *P* = 0.057) (**Figure 8D**).

#### 3.6.5 Subgroup analysis of total hospital stay

In Asia, the total hospital stay in the obese group was significantly shorter (SMD: −0.29; 95% CI: −0.45 to −0.14; *P* = 0.000; I² = 0.0%, *P* = 0.689). In contrast, a statistically non-significant difference was observed between the two groups in the Western Populations (SMD: 0.09; 95% CI: −0.11 to 0.28; *P* = 0.378; I² = 96.3%, *P* = 0.000) (**Figure 8E**).

#### 3.6.6 Subgroup analysis of operation time

In Western regions, operation time in the obese group was significantly longer (SMD: 0.19; 95% CI: 0.11–0.27; *P* = 0.000; I² = 85.3%, *P* = 0.000). In contrast, a statistically non-significant difference was identified between the two groups in Asian populations (SMD: 0.09; 95% CI: −0.33 to 0.52; *P* = 0.662; I² = 71.6%, *P* = 0.060) (**Figure 8F**).

### 3.7 Sensitivity analysis

Sensitivity analysis demonstrated that excluding studies with larger sample sizes—such as Thomas PA (38)—affected the pooled estimate for operation time. Excluding individual studies one at a time did not significantly alter the pooled estimates for postoperative mortality, postoperative complications, pulmonary complications, cardiovascular complications, or total hospital stays, indicating these results were stable and reliable in the meta-analysis (**Supplementary Material 3**).

### 3.8 Publication bias

Funnel plots and Egger’s tests demonstrated no significant publication bias for postoperative mortality, postoperative complications, pulmonary complications, or cardiovascular complications. Due to insufficient data, publication bias was not assessed for other surgical outcomes. Details are provided in **Supplementary Material 4**.

## 4 Discussion

This meta-analysis demonstrates that obese patients undergoing lung cancer surgery experience reduced postoperative mortality, prolonged operative duration, and no increase in postoperative complications or total hospital stays compared with individuals of normal weight.

The concept of the “obesity paradox” has garnered considerable attention across various surgical disciplines, including lung cancer surgery. This paradox posits that contrary to conventional expectations, obesity may confer a survival advantage in certain patient populations. In lung cancer surgery, multiple studies have examined this phenomenon, indicating that patients with obesity may experience reduced postoperative mortality compared to their normal-weight individuals. One study evaluating the impact of BMI on surgical outcomes in patients with NSCLC reported that overweight individuals exhibited slightly improved overall survival relative to those with normal BMI. The finding highlighted preoperative BMI as an independent prognostic factor for survival in patients undergoing complete resection for NSCLC, with underweight patients demonstrating notably poorer survival outcomes (39). A study examining the impact of BMI on mortality rates following lung resection surgery provides further evidence supporting the obesity paradox, revealing that low BMI is significantly associated with poorer postoperative outcomes, including higher 90-day mortality. In contrast, obesity was linked to reduced morbidity and mortality, reinforcing the possibility that higher BMI may exert a protective effect in this surgical setting (40). Additionally, research investigating the role of omentin, an adipokine, in lung cancer suggested that the presence of adipocytes and preadipocytes in normal lung tissue may contribute to the enhanced survival observed in patients with obesity. This study identified omentin as a potential prognostic factor, offering a possible explanation for the obesity paradox in lung cancer (41). In conclusion, the obesity paradox in lung cancer surgery is supported by multiple studies indicating that patients with obesity may experience reduced postoperative mortality compared with their normal-weight counterparts. The present meta-analysis observed decreased postoperative mortality among obese individuals following lung cancer surgery in Western populations. These findings emphasize the complexity of the association between BMI and surgical outcomes and highlight the need for further investigation to fully understand the mechanisms underlying this paradox.

In the context of lung cancer surgery, the association between obesity and postoperative complications has been extensively investigated. A study involving elderly patients undergoing thoracoscopic anatomic lung cancer surgery reported that obesity did not significantly elevate the risk of postoperative complications. However, patients with obesity exhibited higher incidences of intraoperative hypoxemia and new-onset arrhythmia compared with non-obese individuals. Despite these intraoperative events, overall postoperative outcomes, including pulmonary complications, length of hospital stay, and hospitalization costs, did not differ significantly between the two groups (32). Similarly, a study evaluating the impact of obesity on perioperative outcomes in patients undergoing minimally invasive anatomical lung resections found that obesity did not confer early outcome benefits. Although the risk of respiratory complications was higher among patients with obesity, overall, cardiovascular and surgical complication rates did not differ significantly between obese and normal-weight individuals (42). Furthermore, research on the impact of obesity on in-hospital and postoperative outcomes following hepatic resection for malignancy—findings that may be extrapolated to thoracic surgery—indicated a greater risk of complications in open surgeries but not in laparoscopic procedures (43). In patients with obesity and colorectal cancer, laparoscopic surgery was associated with significantly reduced postoperative morbidity and improved healthcare resource utilization compared to open surgery (44). These findings suggest that surgical approaches may help mitigate postoperative risks in patients with obesity. In thoracic surgery for lung cancer, a study investigating the impact of BMI on postoperative nausea and vomiting reported a lower incidence of these complications within the first 48 h among patients with a higher BMI (45). This finding suggests that elevated BMI may not necessarily correlate with increased postoperative complications in certain clinical contexts. Additionally, a study on the ‘obesity paradox’ in minimally invasive anatomical lung resection concluded that obesity did not confer early outcome benefits, as patients with obesity exhibited a significantly higher risk of respiratory complications (42). However, overall complication rates did not differ significantly between patients with obese and normal-weight individuals, reinforcing the notion that obesity does not inherently increase postoperative complications in lung cancer surgery (42).

The research found that obesity in Western patients was associated with prolonged operation duration, although it did not significantly influence postoperative complications or total hospital stays. Obesity complicates surgical procedures due to increased adipose tissue, which can obscure anatomical landmarks and hinder surgical access. This challenge is reflected in the increased operation time observed in patients with obesity undergoing lung cancer surgery. It is well established that body habitus in patients with obesity contributes to technical difficulty during surgery (46). St Julien et al. reported a longer operative time in patients with obesity, with an increase of 7.2 min for every 10-unit rise in BMI (47). Although the present meta-analysis indicates that obesity is associated with increased operative duration, potential confounding by comorbidities such as diabetes and cardiovascular disease must be considered. Many of the included studies did not adjust for these variables, and obesity frequently clusters with metabolic dysfunction, which independently increases perioperative risk. Future research should stratify obesity by metabolic status, such as “metabolically healthy obesity” versus “obesity accompanied by diabetes,” to determine whether the increased operative time is driven by BMI alone or associated metabolic conditions.

Several studies have examined the association between obesity and surgical outcomes in patients with lung cancer. For instance, a study investigating the perioperative outcomes in elderly patients undergoing thoracoscopic anatomic lung cancer surgery reported that those with obesity required longer operative time than non-obese individuals (32). Although obesity has been associated with improved postoperative outcomes in older patients, it may still contribute to greater technical complexity and prolonged surgical duration. Similarly, an analysis of data from the American College of Surgeons National Surgical Quality Improvement Program database reported that patients with obesity undergoing pulmonary resections for lung cancer experienced longer operative times compared to their non-obese counterparts (28). The study emphasized that obesity complicates the surgical process and contributes to extended procedural duration. Moreover, the impact of obesity on operative outcomes extends beyond lung cancer surgery; evidence across multiple surgical disciplines consistently demonstrates that obesity is associated with increased operative times and higher rates of postoperative complications. For example, a study comparing laparoscopic and open surgery outcomes in patients with rectal cancer and obesity found that the laparoscopic approach was associated with reduced blood loss and shorter hospital stays; however, operative time remained longer in patients with obesity compared to those with normal weight (48). Prolonged operative time is a recognized risk factor for complications. As operative duration was significantly extended in patients with obesity, this factor alone may elevate perioperative risk, independent of BMI itself. Overall, these studies highlight the surgical challenges associated with obesity, particularly in lung cancer surgery, where increased operative time is a common consequence. The consistent association between obesity and prolonged operative duration highlights the need for tailored surgical strategies and interventions to improve outcomes in this patient population.

This meta-analysis evaluates the immediate impact of obesity on surgical outcomes in patients with lung cancer, focusing on perioperative metrics such as complications, operative time, length of stay, and mortality. In contrast, long-term oncologic outcomes—including disease-free survival and overall survival—are influenced by many factors beyond surgery, such as adjuvant therapy, tumor biology, and comorbidities, making them less directly attributable to obesity’s perioperative effects. Existing literature on obesity in lung cancer surgery often addresses either perioperative or long-term outcomes but rarely both. Perioperative outcomes are more consistently reported in surgical studies, whereas long-term survival data require extended follow-up, which is often unavailable.

This meta-analysis focused on observational studies for several key reasons. Existing randomized controlled trials typically compare surgical techniques or perioperative care protocols rather than obesity as an exposure, making them less relevant to the research objective. Observational studies more accurately reflect routine clinical practice, in which patients with varying BMI levels undergo surgery without randomization. This design aligns with the aim of evaluating the real-life impact of obesity on perioperative outcomes. Most available evidence on obesity and surgical outcomes derives from retrospective or prospective cohort studies. Consequently, most surgical meta-analyses examining patient-specific factors, such as BMI or age, rely on observational data.

This meta-analysis offers several key strengths. First, this is the first meta-analysis to evaluate the effect of obesity on perioperative outcomes in patients undergoing lung cancer surgery, addressing a significant gap in the literature and providing novel insights into the association between obesity and surgical outcomes. Second, this meta-analysis aggregates data from multiple studies, offering a more robust and statistically powerful assessment of the impact of obesity on perioperative outcomes than any individual studies. Third, the findings may inform clinical decision-making by highlighting the potential risks and benefits of lung cancer surgery in patients with obesity, thereby supporting preoperative planning and patient counseling. Moreover, sensitivity analyses were performed to assess the stability of the results, ensuring that no single study or outlier disproportionately influenced the findings. Publication bias was evaluated using Egger’s test and funnel plots, enhancing the credibility of the meta-analysis by addressing potential reporting biases. Due to insufficient reporting of outcomes stratified by surgical approach in the included studies, subgroup analyses could not be performed. However, sensitivity analyses excluding studies dominated by open or minimally invasive techniques were conducted to assess the robustness of pooled estimates. Although VATS and open surgery differ in invasiveness, obesity presents shared challenges in both approaches, including prolonged anesthesia time and technical difficulties related to body habitus. Therefore, the pooled estimates reflect the aggregate risk across real-world practices. Ultimately, the inherent variability in defining postoperative complications across healthcare systems and geographic regions was addressed; therefore, a random-effect model was applied to account for heterogeneity, offering a more conservative estimate.

However, this meta-analysis has some limitations. First, it may not fully account for all relevant factors influencing perioperative outcomes, such as comorbidities, smoking status, or variations in postoperative care, which may confound the results. As a research-level meta-analysis, the analysis is constrained by the variables reported in the original studies. Due to limited data on smoking history, lung function, or nutritional status, these patient-level variables were not included. Second, due to insufficient data, the meta-analysis focused primarily on short-term perioperative outcomes, potentially overlooking the long-term effects of obesity on recovery, survival, and quality of life after lung cancer surgery. Despite these limitations, this meta-analysis provides valuable insights and establishes a foundation for future research into the impact of obesity on perioperative outcomes in lung cancer surgery. Last, combining varying BMI thresholds used for Asians and Western populations into a single “obesity” category increases heterogeneity. Although this binary classification of obesity may oversimplify the association between BMI and surgical risk, it was necessitated by inconsistent reporting across studies. Future investigations should adopt standardized BMI stratification to identify potential threshold effects and better quantify risk gradients.

## 5 Conclusion

This meta-analysis suggests that the obesity paradox may indeed exist in patients undergoing lung cancer surgery. While obesity is generally associated with prolonged operative duration, it does not appear to increase postoperative complications or total hospital stays and is often linked to reduced mortality. The prognostic role of obesity has been confirmed; however, potential racial differences may limit the generalizability of these findings across clinical settings and remain insufficiently explained. Consequently, these results require validation and refinement through well-designed, international studies. Future research should focus on disentangling obesity phenotypes, such as sarcopenic obesity, refining outcome predictors, and integrating genomic and metabolic data to enhance understanding of individual risk profiles.

## Data Availability

The original contributions presented in the study are included in the article/**Supplementary Material**. Further inquiries can be directed to the corresponding authors.
