# Supplementary material for: Effect of obesity on perioperative outcomes following lung cancer surgery: a systematic review and meta-analysis
Source: Front Oncol. 2025 Sep 25;15:1600503. doi: 10.3389/fonc.2025.1600503 (PMC12507620; doi:10.3389/fonc.2025.1600503)
Supplement: Supplementary file 2 [file DataSheet2.docx]

| [Supplementary material 1](https://pmc.ncbi.nlm.nih.gov/articles/PMC9609787/#DS1) Search strategy in PubMed database |
| --- |
| Search items |
| #1 ((((((((((((Lung Neoplasms[MeSH Terms]) OR (Neoplasms, Pulmonary[Title/Abstract])) OR (Neoplasm, Pulmonary[Title/Abstract])) OR (Pulmonary Neoplasm[Title/Abstract])) OR (Lung Neoplasm[Title/Abstract])) OR (Lung Cancer[Title/Abstract])) OR (Lung Cancers[Title/Abstract])) OR (Cancer of Lung[Title/Abstract])) OR (Pulmonary Cancer[Title/Abstract])) OR (Cancer, Pulmonary[Title/Abstract])) OR (Cancers, Pulmonary[Title/Abstract])) OR (Pulmonary Cancers[Title/Abstract])) OR (Cancer of the Lung[Title/Abstract])  #2 ((((((Thoracic Surgery, Video-Assisted[MeSH Terms]) OR (Video-Assisted Thoracic Surgeries[Title/Abstract])) OR (VATS[Title/Abstract])) OR (VATSs[Title/Abstract])) OR (Video Assisted Thoracic Surgery[Title/Abstract])) OR (Video-Assisted Thoracoscopic Surgeries[Title/Abstract])) OR (Video Assisted Thoracoscopic Surgery[Title/Abstract])  #3 (pulmonary surgical procedures[MeSH Terms]) OR (Pulmonary Surgical Procedure[Title/Abstract])  #4 #2or #3  #5 ((Body Mass Index[MeSH Terms]) OR (Quetelets Index[MeSH Terms])) OR (Quetelet Index[MeSH Terms])  #6 Obesity[MeSH Terms]  #7 #5 or #6  #8 #1and #4 and #7 |
