# Supplementary material for: Effect of obesity on perioperative outcomes following lung cancer surgery: a systematic review and meta-analysis
Source: Front Oncol. 2025 Sep 25;15:1600503. doi: 10.3389/fonc.2025.1600503 (PMC12507620; doi:10.3389/fonc.2025.1600503)
Supplement: Supplementary file 3 [file DataSheet3.docx]

[Supplementary material 2](https://pmc.ncbi.nlm.nih.gov/articles/PMC9609787/#DS1):Sensitivity analysis


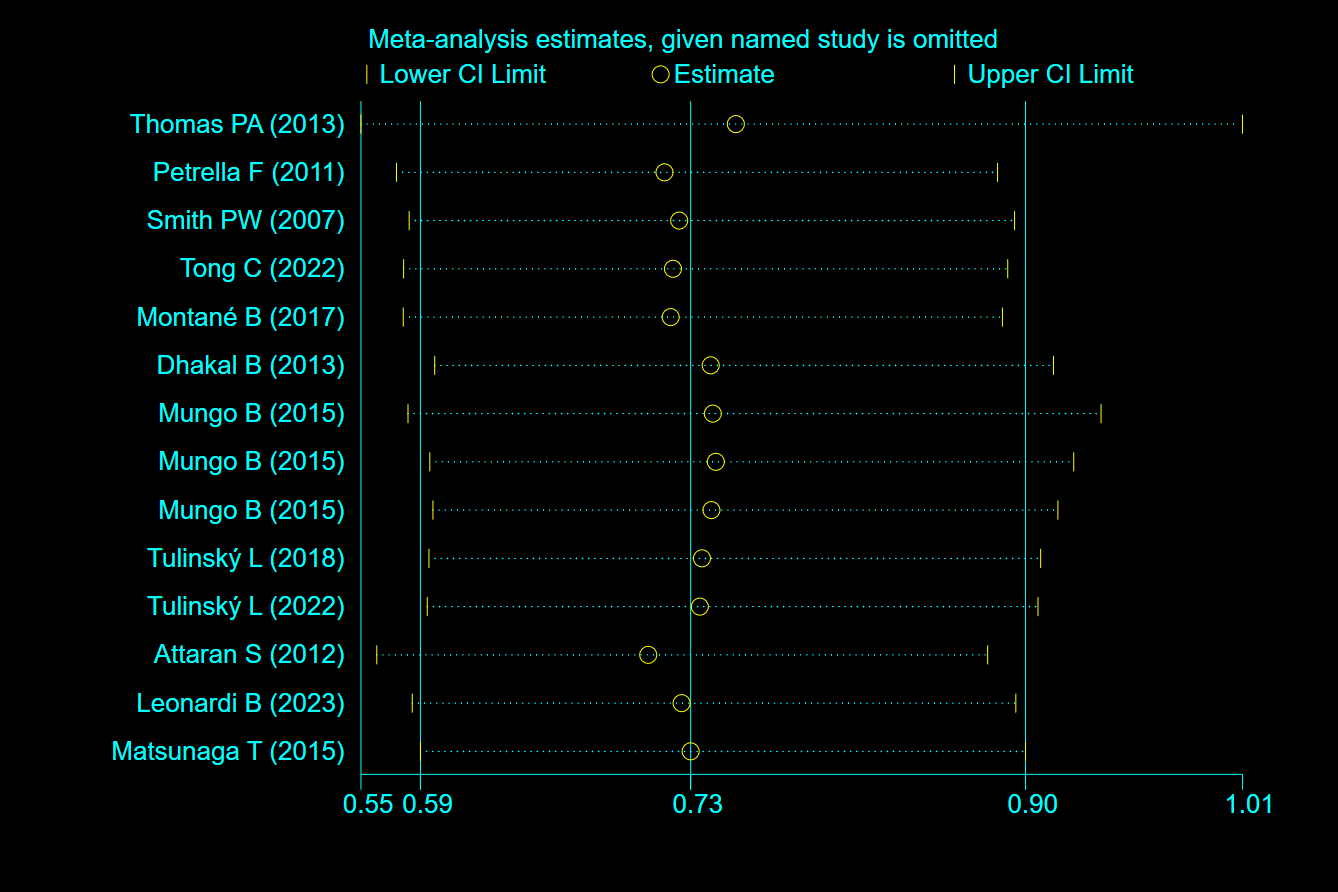


**Figure 1.** Sensitivity analysis of **the postoperative mortality**

**
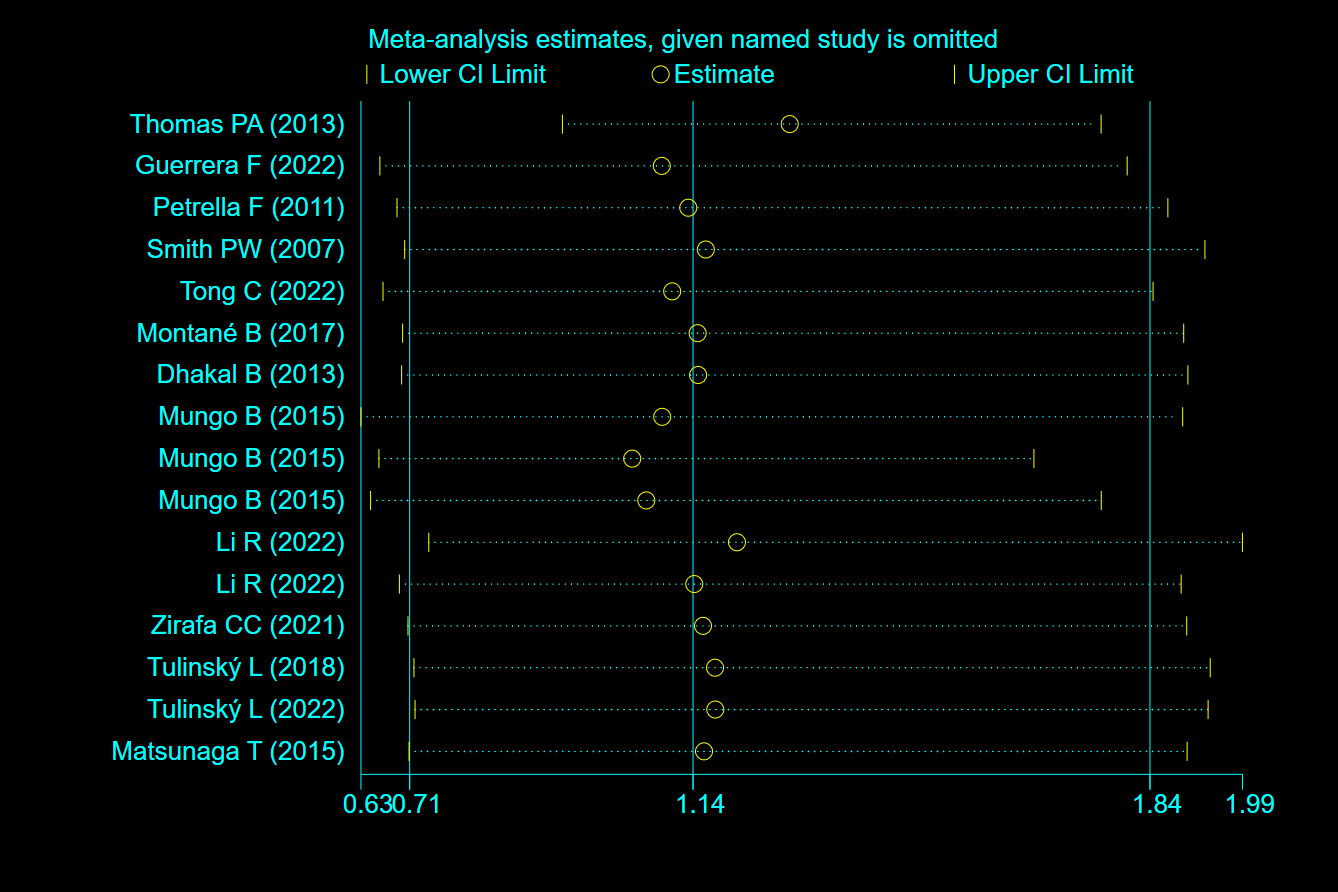
**

**Figure 2.** Sensitivity analysis of **the postoperative complications**

**
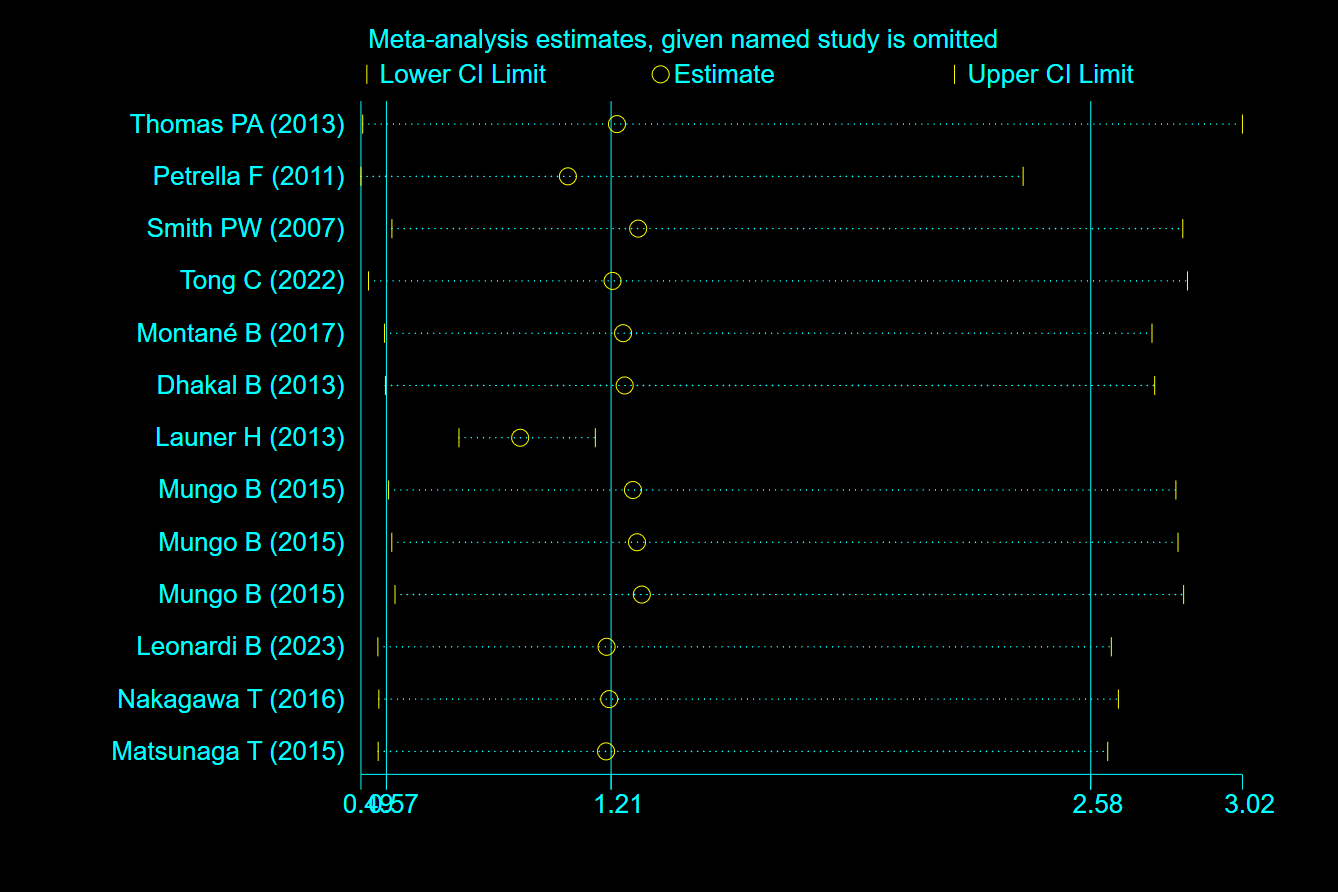
**

**Figure 3.** Sensitivity analysis of **the pulmonary complications**

**
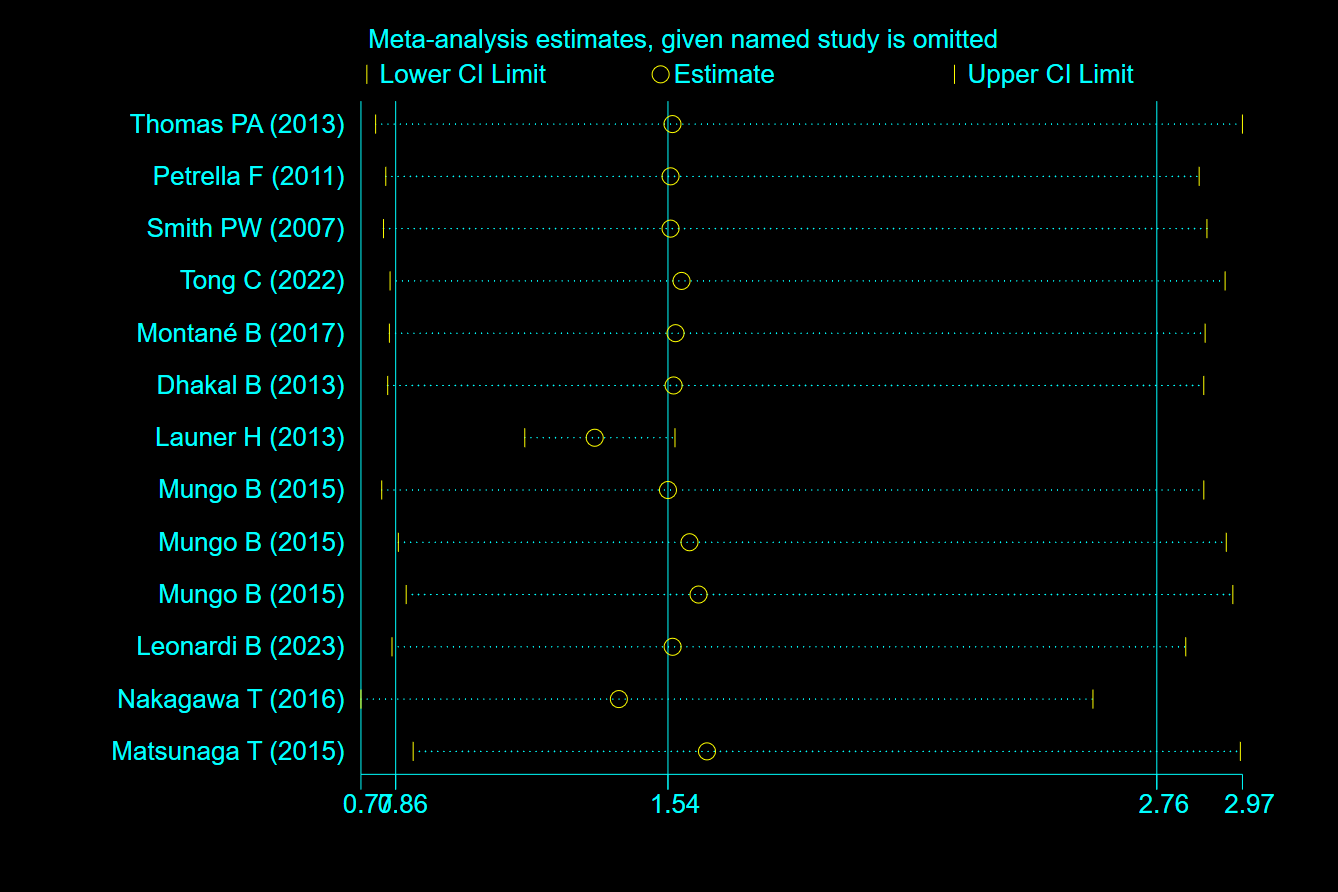
**

**Figure 4.** Sensitivity analysis of **the cardiovascular complications**

**
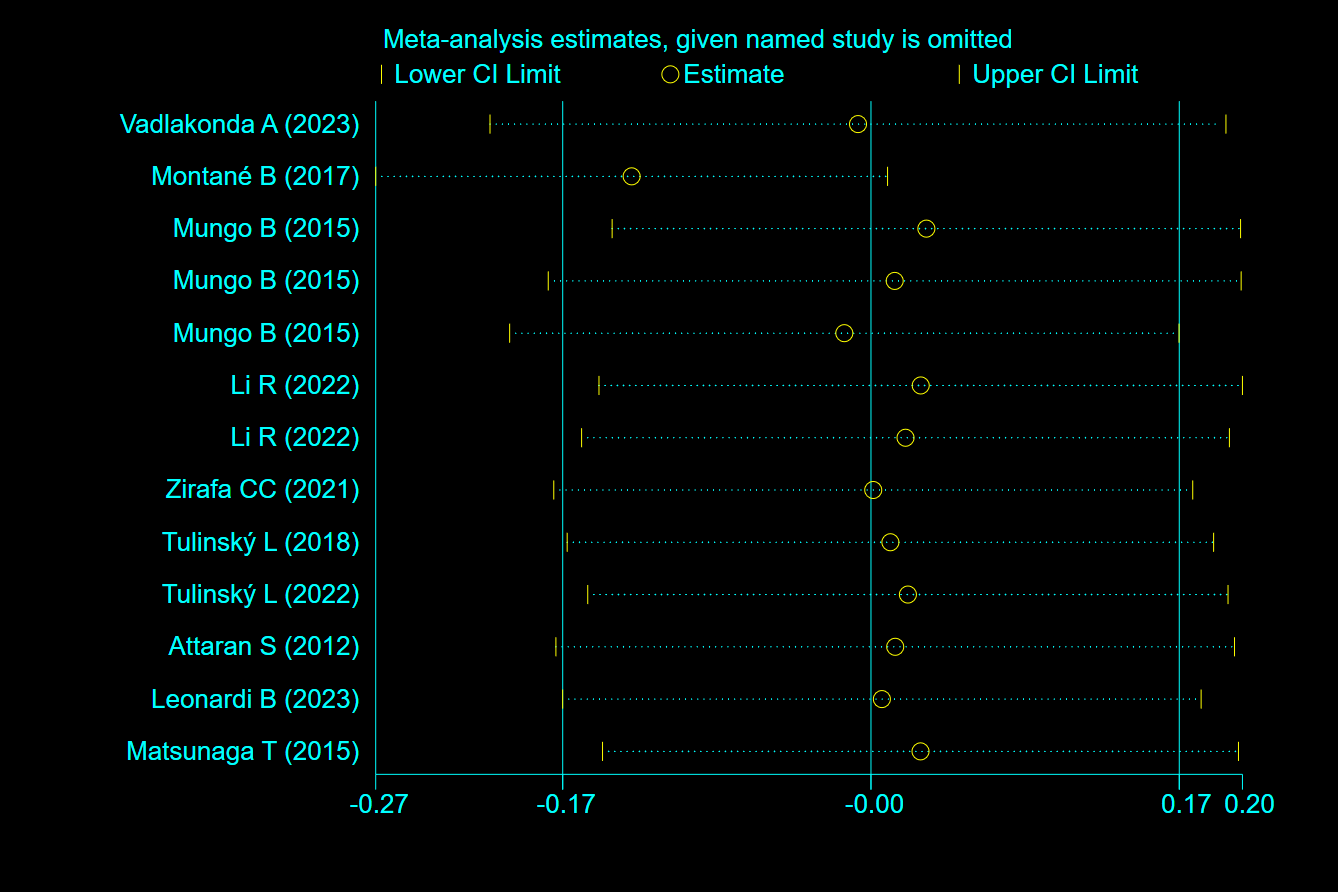
**

**Figure 5.** Sensitivity analysis of **the total hospital stays**

**
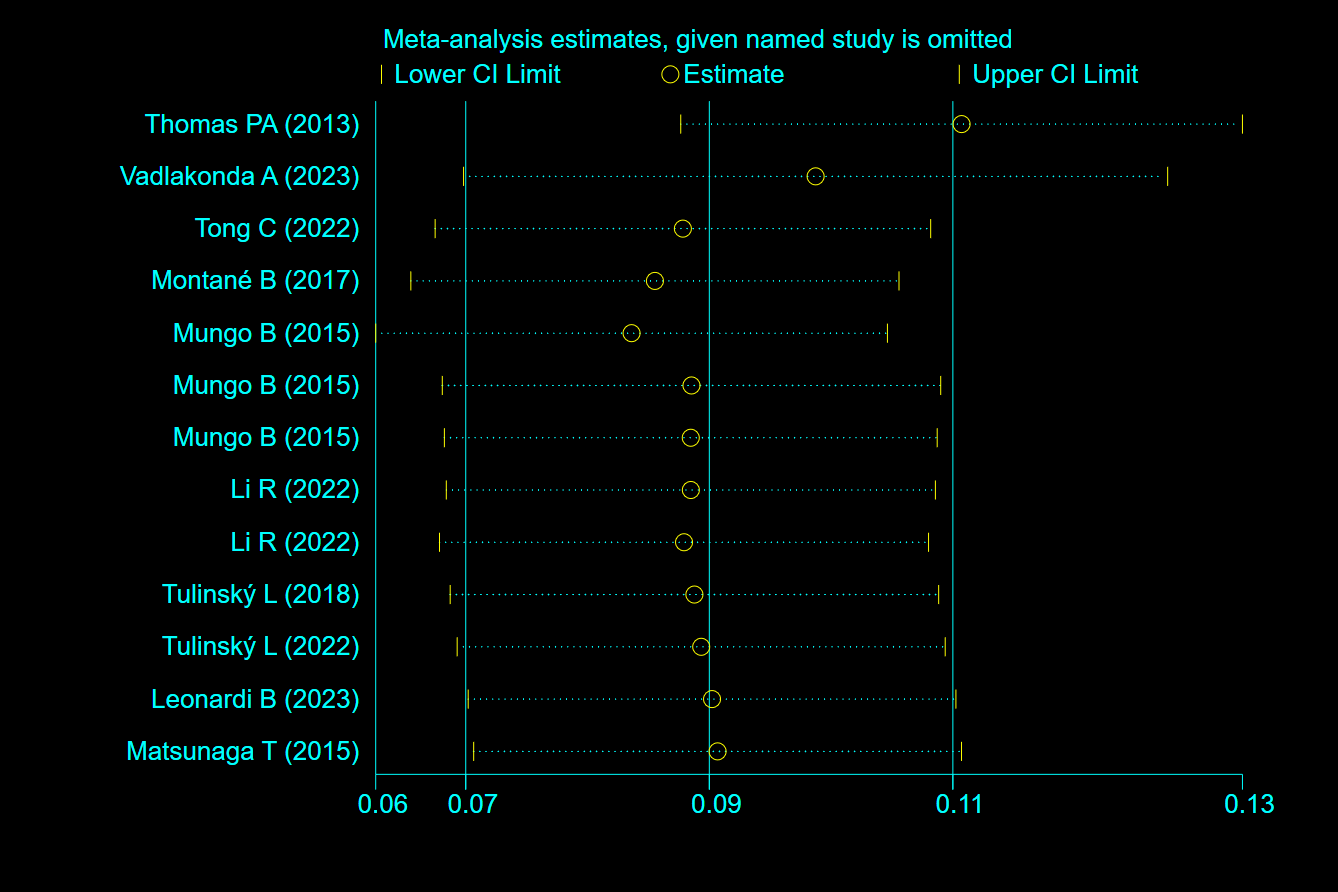
**

**Figure 6.** Sensitivity analysis of **the operation time**
