# Supplementary material for: Effect of obesity on perioperative outcomes following lung cancer surgery: a systematic review and meta-analysis
Source: Front Oncol. 2025 Sep 25;15:1600503. doi: 10.3389/fonc.2025.1600503 (PMC12507620; doi:10.3389/fonc.2025.1600503)
Supplement: Supplementary file 4 [file DataSheet4.docx]

Supplementary material 3：The results of funnel plots and Egger’s tests

**
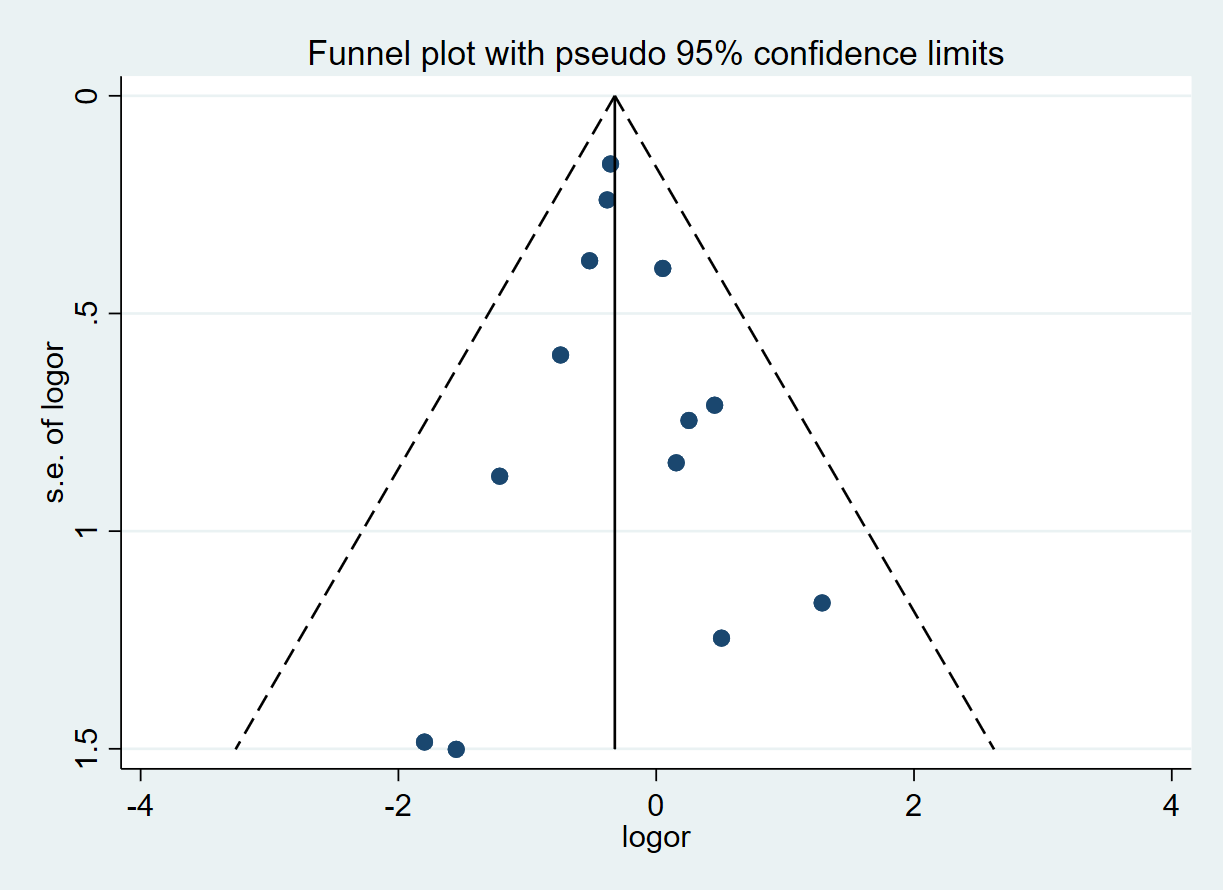
**

**Figure 1.** Funnel plot of **postoperative mortality** (Egger^,^s test, p=P=0.670)


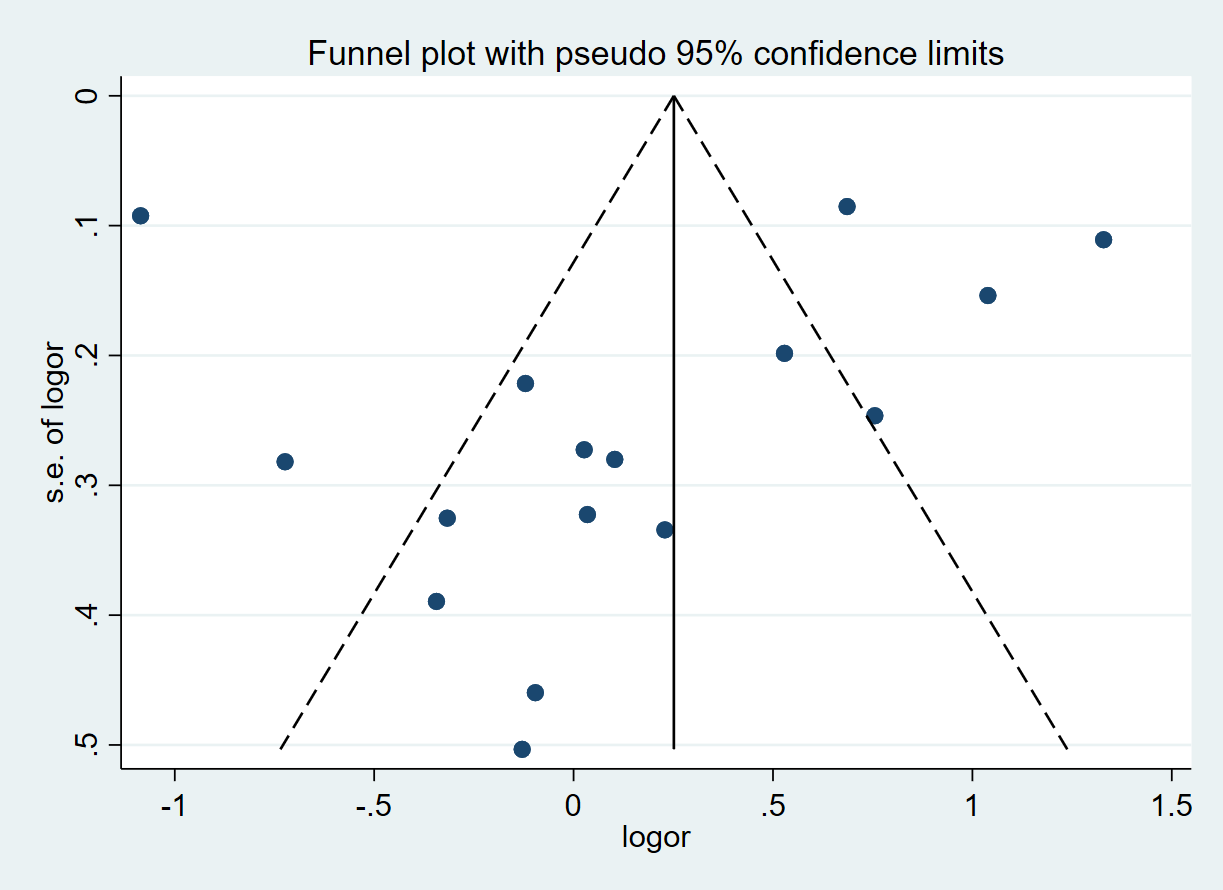


**Figure 2.** Funnel plot of **postoperative complications** (Egger^,^s test, p=0.777)


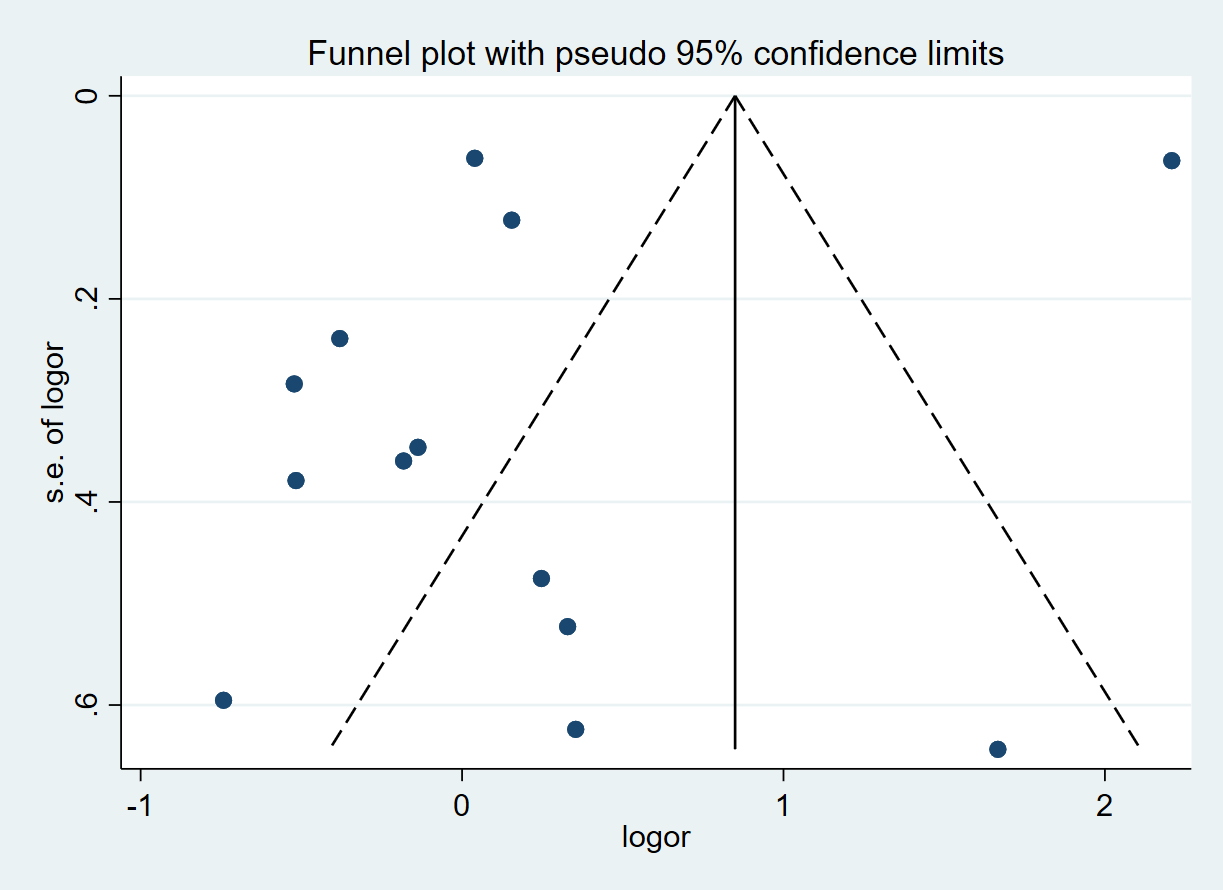


**Figure 3.** Funnel plot of **pulmonary complications** (Egger^,^s test, p=0.308)


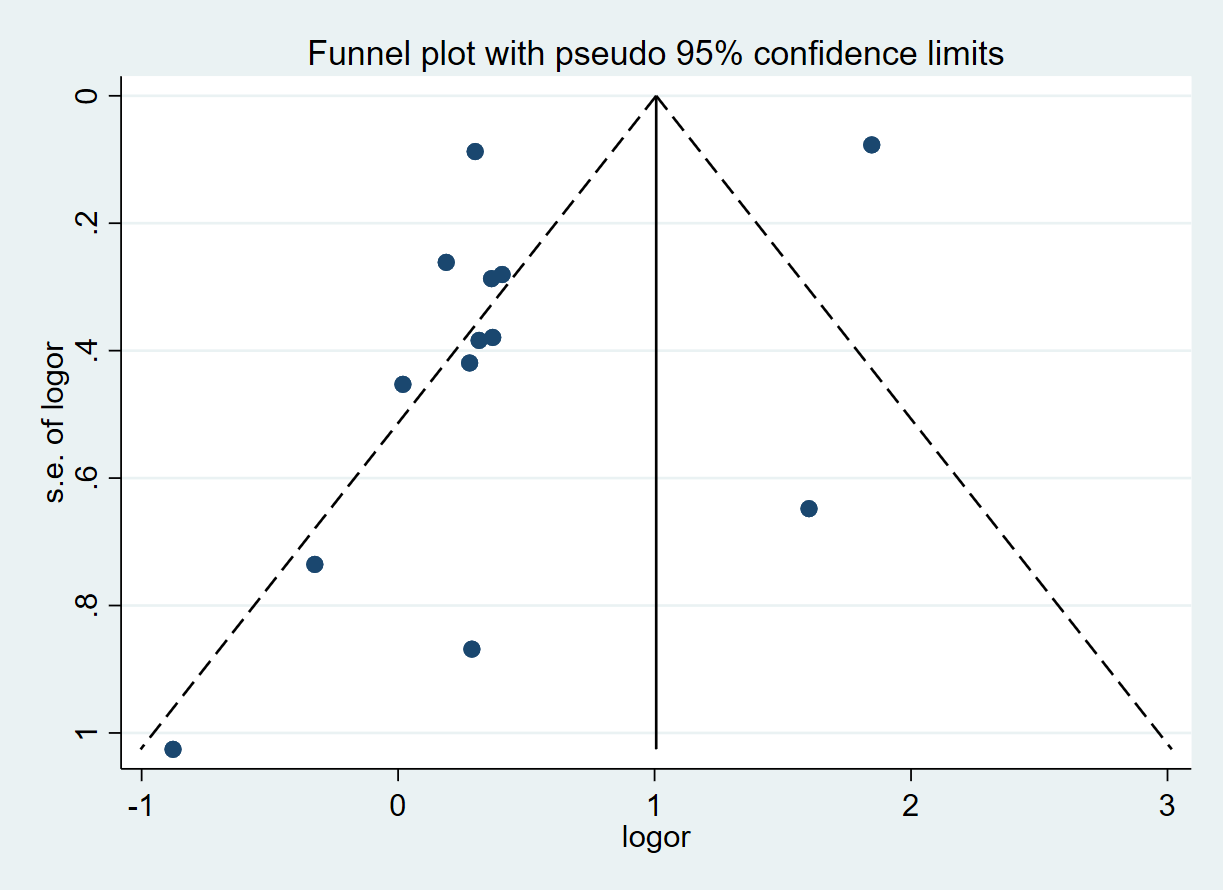


**Figure 4.** Funnel plot f of **cardiovascular complications** (Egger^,^s test, p=0.155)
